# The behavioral sensitivity of mice to acyclic, monocyclic, and bicyclic monoterpenes

**DOI:** 10.1371/journal.pone.0298448

**Published:** 2024-02-23

**Authors:** Ellie Williams, Austin Pauley, Adam Dewan

**Affiliations:** Department of Psychology, Program in Neuroscience, Florida State University, Tallahassee, FL, United States of America; Universidade Federal do Para, BRAZIL

## Abstract

Monoterpenes are a large class of naturally occurring fragrant molecules. These chemicals are commonly used in olfactory studies to survey neural activity and probe the behavioral limits of odor discrimination. Monoterpenes (typically in the form of essential oils) have been used for centuries for therapeutic purposes and have pivotal roles in various biological and medical applications. Despite their importance for multiple lines of research using rodent models and the role of the olfactory system in detecting these volatile chemicals, the murine sensitivity to monoterpenes remains mostly unexplored. We assayed the ability of C57BL/6J mice to detect nine different monoterpenes (the acyclic monoterpenes: geraniol, citral, and linalool; the monocyclic monoterpenes: r-limonene, s-limonene, and γ-terpinene; and the bicyclic monoterpenes: eucalyptol, α-pinene, and β-pinene) using a head-fixed Go / No-Go operant conditioning assay. We found that mice can reliably detect monoterpene concentrations in the low parts per billion (*ppb*) range. Specifically, mice were most sensitive to geraniol (threshold: 0.7 ppb) and least sensitive to γ-terpinene (threshold: 18.1 ppb). These estimations of sensitivity serve to set the lower limit of relevant monoterpene concentrations for functional experiments in mice. To define an upper limit, we estimated the maximum concentrations that a mouse may experience in nature by collating published headspace analyses of monoterpene concentrations emitted from natural sources. We found that natural monoterpenes concentrations typically ranged from ~1 to 1000 ppb. It is our hope that this dataset will help researchers use appropriate monoterpene concentrations for functional studies and provide context for the vapor-phase delivery of these chemicals in studies investigating their biological activity in mice.

## Introduction

Monoterpenes are a large class of volatile metabolites produced by plants in order to attract pollinators, reduce the impact of biotic / abiotic stressors, and even trigger an immune response in distant plants to combat herbivory [1, 2]. Their low molecular weight and high lipophilicity confer high absorption and membrane penetration capacities contributing to their biological action and medicinal potential [3, 4]. Specifically, monoterpenes (and their derivatives) have been shown to have analgesic, antioxidant, and anti-inflammatory properties in humans [2, 5] as well as antioxidant, sedative, antinociceptive, and anxiolytic-like effects in mice [6–12], and have been widely studied for their potential as natural insecticides [13, 14]. Monoterpenes may also act as signaling molecules—influencing energy metabolism with anti-diabetic and anti-obesity properties [15]. Beyond these medicinal applications, monoterpenes are also prominent components of the flavor of many fruits and vegetables [16] and thus contribute to ingestive behavior. Despite the clear importance of monoterpenes for multiple lines of research using rodent models and the role of the olfactory system in detecting these volatile chemicals, the murine sensitivity to monoterpenes remains mostly unexplored.

The goal of this study was to define the detection threshold of mice to volatile odorants within each monoterpene division, specifically geraniol, citral, and linalool (acyclic monoterpenes); r-limonene, s-limonene, and γ-terpinene (monocyclic monoterpenes); and eucalyptol, α-pinene, and β-pinene (bicyclic monoterpenes). Previous studies have estimated murine sensitivity for only three of these compounds (eucalyptol, r-limonene, and s-limonene) [17–19]. Even among this limited odor set, our understanding of murine sensitivity remains clouded, as the only chemical tested in multiple studies (r-limonene) has published thresholds that differ by ~15 orders of magnitude [17, 18]. Our established behavioral method has resulted in estimates of olfactory sensitivity that are internally consistent, across both individuals and different cohorts even when tested in different behavioral rigs by different experimenters [20–24]. Our results will complement these previous studies by providing the first estimation of sensitivity for six of these monoterpenes and help clarify the large discrepancy between published monoterpene thresholds in mice.

Monoterpenes are also commonly used in olfactory research to survey neural activity in a variety of olfactory regions, probe the behavioral limits of odor discrimination, and assess odor-structure activity relationships in mice [17, 25–32]. The stimulus intensities used in these studies vary widely as appropriate concentrations have yet to be defined. Our estimations of monoterpene sensitivity can help define the low-end of relevant monoterpene concentrations that should be used in functional experiments. To define the high-end of relevant concentrations, we have combed the literature for headspace analyses of monoterpenes emitted from natural sources. Together, these data will hopefully guide researchers in choosing appropriate stimulus concentrations for functional experiments utilizing monoterpenes.

## Materials and methods

The methods utilized in this study are well documented [20–23], including a step-by-step guide to our surgical approach, behavioral training, odor delivery, and operant conditioning assay [24].

### Animals

Male and female C57BL/6J mice (16 M; 16 F) were housed in same-sex cages until head-bar surgery. Mice (10–14 weeks old) were anesthetized with isoflurane at a dosage of 2–3% in oxygen, administered buprenorphine ER (0.1 mg/kg) as an analgesic, and lidocaine (2 mg/kg) as a local anesthetic. A custom head-bar was affixed to the skull using 2–3 micro-screws and secured using dental cement. After surgery, mice were individually housed and given at least three days to recover. Following recovery, mice were water restricted for at least two weeks before training in a water-rewarded conditioning paradigm [24]. At the conclusion of the experiment, mice were euthanized via carbon dioxide inhalation. All procedures conducted were reviewed and approved by the Florida State University Animal Care and Use Committee [Protocol # 202100020]. All efforts were made to ensure the health and welfare of the animal throughout the experiment.

### Odor stimuli

A set of nine monoterpenes was used in this study, including three acyclic monoterpenes: linalool (CAS# 78-70-6), geraniol (CAS# 106-24-1), and citral (CAS# 141-27-5); three monocyclic monoterpenes: r-limonene (CAS# 5989-27-5), s-limonene (CAS# 5989-54-8), and γ-terpinene (CAS# 99-85-4); and three bicyclic monoterpenes: α-pinene (CAS# 80-56-8), β-pinene (CAS# 127-91-3), and eucalyptol (CAS# 470-82-6). The use of both isomer (α-pinene / β-pinene) and enantiomer (r-limonene / s-limonene) pairs allowed for a comparison of mouse sensitivity across structurally similar molecules. All odorants were obtained from Millipore-Sigma at the highest available purity (>98%), stored under nitrogen, and housed in a chemical storage cabinet. Odorants were diluted with mineral oil (CAS# 8042-47-5) within an odor-free chemical safety cabinet with the use of filtered pipette tips. The maximum odorant concentration tested was a 2:100 dilution (or 2% v/v) for all odorants.

To determine if behavioral sensitivity was correlated with specific odorant features, we obtained/calculated the air/mucus odorant partition coefficient, volatility, and atmospheric lifetime for each odorant. The air/mucus odorant partition coefficient is defined by the ratio of odorant concentration in the air phase to the concentration in the mucus at the air-mucus interface [33]. The air/mucus odorant partition coefficient (β) was calculated based on the following equation [33]:

$$log\beta =log\beta_{water}-(logP-1)\times 0.524$$

where β_water_ is the air/water partition coefficient and P is the octanol/water partition coefficient, both of which were obtained through U.S. Environmental Protection Agency database [Estimation Program Interface [Epi] Suite tool; www.epa.gov/oppt/exposure/pubs/episuite.htm]. Atmospheric chemical lifetime and volatility measures for each odorant were also obtained through [Epi] Suite tool (EPA). Atmospheric lifetime is a measure of the rate of oxidation for an odorant and was previously correlated to human olfactory sensitivity [34].

The vapor-phase concentration for eight of the odorants, were determined according to published equilibrium equations [35]. The relationship between the liquid- and vapor-phase concentration of citral in mineral oil was determined using a photoionization detector (PID) based approach [36]. Briefly, we measured the net charge resulting from the ionization of the headspace above pure and diluted citral concentrations using a calibrated PID with the use of a correction factor. To calculate the correction factor for citral, a sealed bottle containing a small volume of this odorant (2.5μl and subsequently verified with 5.0 μl) was heated beyond its boiling point. With the use of three-way valves, a mass flow controller (MFC) directed the vaporized sample from the temporarily sealed bottle to the PID. The vapor concentration (within the bottle) was divided by the equivalent isobutylene concentration (as determined by the PID voltage) to calculate the correction factor for that odorant. This resulted in a correction factor of 2.05 and a saturated vapor concentration of 112 ppm for citral. This measurement closely matched the predicted vapor concentration of 120 ppm as calculated by the following equation:

$$C\;(ppm)=\frac{P_{vap}}{P_{atm}}(10^{6})$$

Where C is the concentration in ppm, *P*_*vap*_ is the vapor pressure of citral (0.0193 mmHg), and *P*_*atm*_ is the atmospheric pressure in mmHg at 25°C. To estimate the relationship between the liquid and vapor-phase concentration of citral, five PID measurements were taken from each liquid dilution with a 30 second inter-trial interval. The calibrated net charge for each trial was multiplied by the citral correction factor to determine the vapor-phase concentration resulting from the associated liquid dilution. These data were plotted and fitted with a power function ([C]_vap_ = a[C]_liq_^β^; Prism Graphpad).

$${\left[citral\right]}_{vap}=26.3*{{\left[citral\right]}_{MO}}^{0.90}.$$

To estimate the solvent-corrected vapor-phase threshold of a published study [17] testing murine sensitivity to r-limonene and s-limonene in diethyl phthalate, we measured the correction factors and equilibrium equations associated with these odorants in diethyl phthalate using the same method as above. Our analysis yielded a correction factor of 0.72 for both odorants and the following equilibrium equations:

$${\left[s‐limonene\right]}_{vap}=82.7*{{\left[s‐limonene\right]}_{DEP}}^{0.92}$$

$${\left[r‐limonene\right]}_{vap}=95.9*{{\left[r‐limonene\right]}_{DEP}}^{0.90}$$

Our estimation of the saturated vapor concentration of these odorants (r-limonene: 1924 ppm and s-limonene: 1932 ppm) was very similar to the predicted vapor concentration of 1907 ppm.

### Stimulus delivery

**Fig 1:**
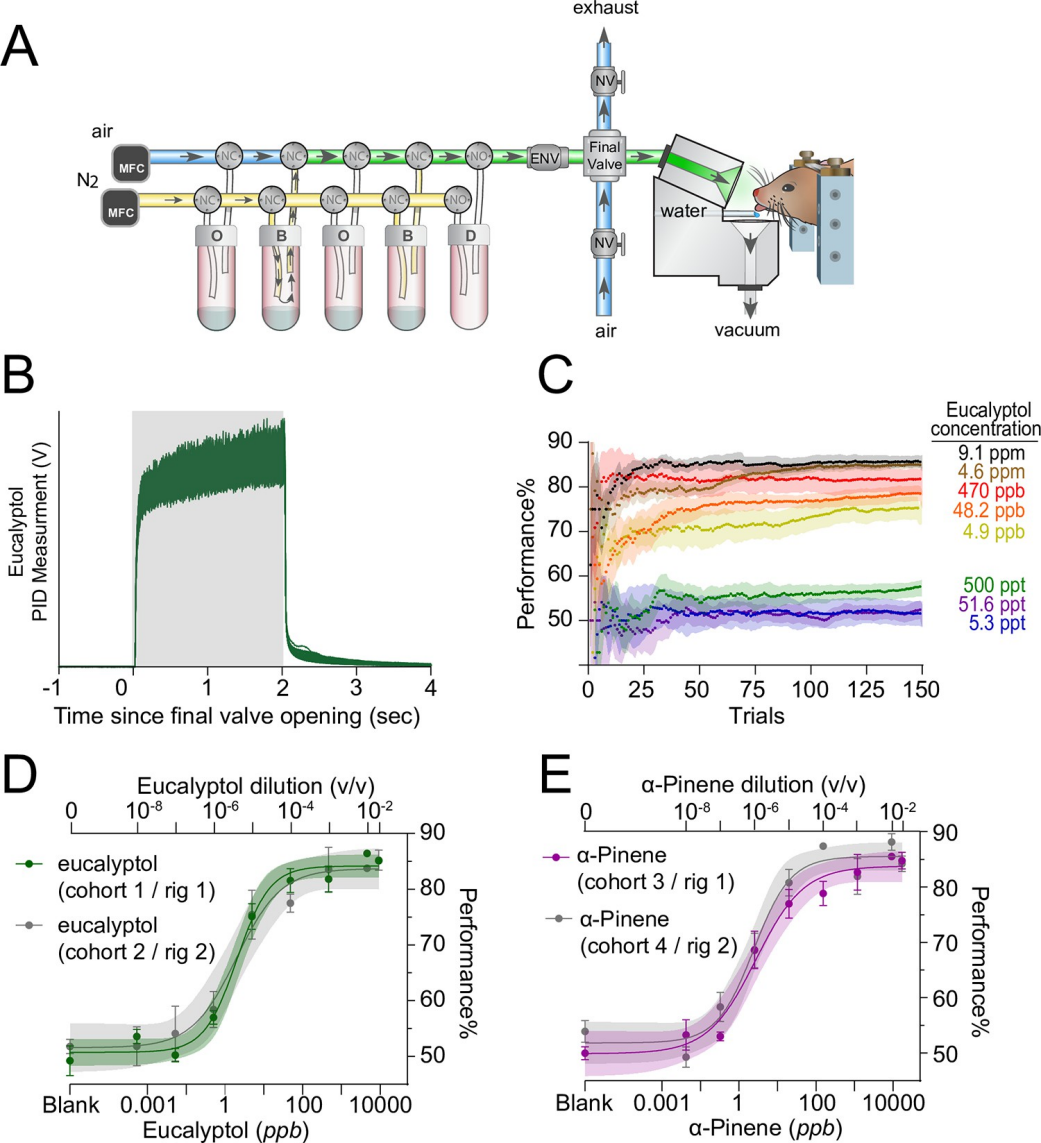
To measure behavioral detections thresholds, we used a head-fixed Go / No-Go operant conditioning assay. (**A**) Odorants were delivered using an 8-channel flow dilution olfactometer with mass flow controllers (MFC) that switch between a pressure-balanced dummy (D) vial (via normally open valves, NO) and either odor (O) or solvent-only (B) vials (via normally closed valves, NC). Odorized air is directed to exhaust to allow the stimulus to reach equilibrium prior to stimulus delivery. During stimulus application, a final valve (dual-synchronous solenoid valve) redirects pressure-balanced, odorized air from exhaust to the animal. Manual needle valves (NV) are used to pressure-balance the air and exhaust lines, while an electronic valve (ENV) is used to pressure-balance individual vials across the olfactometer manifold. At the conclusion of the trial, the final valve returns the pressure-balanced clean air to the animal. (**B**) Photoionization detector (PID) traces of 250 stimulus presentations of eucalyptol. Shaded area signifies two second stimulus period. (**C**) Average cumulative behavioral performance across 150 trials for all concentrations of eucalyptol. Initial go trials are not included. Line signifies mean with shaded SE. (**D-E**) Our experimental approach did not differ across mouse sub-cohorts (N = 4) or different behavioral setups / olfactometers. Data were fitted using a Hill function. Maximal behavioral performance for each odorant concentration is limited to ~85% (see methods). Plots show mean +/- SE with shaded 95% confidence interval.

Disposable 40 mL amber glass vials filled with 15 mL of diluted odorant (or solvent) were attached to the 8-channel olfactometer (Fig 1A) and pressurized before the start of the first trial. Nitrogen gas regulated by a 100 mL/min MFC flowed through the selected vial before it was diluted 10 times by the main air flow stream (regulated by a 900 mL/min air MFC). During stimulus delivery, the final valve swapped the flow to the animal from clean air to diluted odorant. The selected vial is actuated 0.6-s before stimulus delivery to allow the odor concentration to reach equilibrium before delivery to the animal. The odor port is attached to a micromanipulator to standardize the distance to the nose for each head-fixed animal. To verify the stability and reproducibility of our odorant presentations, a PID was used in place of the mouse. A single vial containing 15 mL of the diluted odorant was repeatedly actuated (250 times) with a 15-s inter-trial interval (Fig 1B and S1 Fig).

### Behavioral assay

Water-restricted mice were trained to report the detection of odor in a Go/No-Go task [20–24]. Each cohort of mice initially consisted of four males and four females (age-matched). Cohorts were tested on a maximum of three odorants to limit over-training and the probability that mice would learn to solve the task using non-odor cues (see below). Individual mice were excluded from the experiment if they failed to reach the training criterion for a particular odorant (n = 4 out of 32) or learned to solve the task using non-odor cues (n = 0 out of 32).

Behavioral training consisted of two stages. Stage 1—the mice were trained to receive a water reward if they licked during the stimulus period. Stage 2—mice were trained in a Go / No-Go odor detection task. Sessions typically lasted 120–300 trials and were terminated after the mice missed three Go trials in a row or the mouse reached 300 trials. Behavioral performance was determined by the number of correct responses (hits + correct rejections) divided by the total number of trials (after the initial Go trials). Upon reaching criterion (two sessions >90% correct), mice were subsequently tested in the thresholding assay. Mice that did not reach criterion in a maximum of 4 days were excluded.

To determine behavioral thresholds, mice were only tested on one concentration per day. This approach eliminated any masking/adaptation effects resulting from the contamination of the olfactometer by higher concentrations of the target odor. The olfactometer was loaded with three solvent (Go) vials, three diluted odor (No-Go) vials, and a single solvent (No-Go) vial. Each vial was replaced daily, and their positions were randomized. The solvent No-Go vial (or “cheating check”) served to test whether the mice were using cues other than the presence or absence of the target odor to maximize performance. This solvent No-Go vial should be indistinguishable from other solvent-only Go vials unless the animal is using non-odor cues to maximize performance and the associated water reward. Thus, mice are “cheating” at this task if they are able to reject (i.e. not lick) the solvent No-Go vial at a frequency higher than the percentage of misses (i.e. not licking during a solvent Go vial). If this occurred, the session was excluded from the analysis. If this occurred multiple times (i.e., > 2) over the course of an experiment, the mouse was removed from the experimental group. Since this check is included in our thresholding analysis, the maximum performance a mouse can attain using only odor cues in this experiment is approximately 85% (in contrast to stage 2 training in which the mice can achieve 100% behavioral performance). After the completion of all odorant concentrations, the mouse’s ability to discriminate between vials using non-odor cues was again tested by loading the olfactometer with only solvent vials. These data are included in each figure.

At the end of each day, the olfactometer (including the manifolds and all tubing) was flushed with acetone, 70% isopropanol, and nanopure water, and then was dried with pressurized clean air overnight. The vial caps and tubing were also cleaned with acetone, isopropanol, and nanopure water, and then dried overnight.

### Statistical analysis

Behavioral performance for each odorant was fitted with a four-parameter Hill equation (Prism Graphpad).

$$R=R_{min}+C^{n}*\frac{R_{max}-R_{min}}{\left[C^{n}+C_{½}^{n}\right]}$$

where *R* is the behavioral accuracy, *C* is odor concentration, *C*_½_ is the concentration at half-maximal performance, and *n* is the Hill coefficient. The goodness-of-fit for odorant-specific Hill equation parameters were compared with a sum-of-squares F test (Prism Graphpad). Behavioral thresholds (*C*_½_) were compared between odorants and sexes using a two-way ANOVA with multiple comparisons (Prism GraphPad).

### Human thresholds and source concentrations

To contextualize our estimations of murine sensitivity, we collated published measures of human sensitivity and monoterpene concentrations emitted from natural sources. Estimations of human sensitivity were obtained from Gemert, 2011 [37] and consisted of 72 monoterpene thresholds from 37 sources. Estimations of natural source concentrations were taken from published studies utilizing either headspace volatile analysis via gas chromatography/mass spectrometry (GC/MS) or proton transfer reaction mass spectrometry (PTR-MS). This literature review resulted in data from 22 studies analyzing the headspace volatile concentrations of monoterpenes emitted from 37 compounds. Importantly, we limited our analysis to sources for which 1) mice could reasonably experience and 2) methods in which the sample was not modified by solvent extraction methods. Please note that the PTR-MS method is limited in its ability to unambiguously identify compounds with the same mass/charge ratio. In such cases where a volatile compound was identified as one of a couple possibilities (e.g., r-limonene or s-limonene), the concentration value was assigned to both odorants. Our literature review included headspace volatile analyses from several vegetables (carrot, tomato, and cucumber [38–41]), fruits / fruit juices (blueberry, peach, tangerine, tangerine peel, grapefruit, strawberry, strawberry sepals, orange juice, apple, quince, litchi, and loquat [40, 42–50]), spices (saffron, black and white pepper, cinnamon, and nutmeg [51]), and several other items (tea, almonds, cocoa beans, animal urine, bread, olive oil, truffles, and pine needles [40, 52–59]).

## Results

To measure behavioral detection thresholds, we used a head-fixed Go / No-Go operant conditioning assay (Fig 1A). Our stimulus delivery system resulted in consistent odor pulses throughout a session (Fig 1B and S1 Fig). As shown previously, behavioral performance at each concentration can be predicted after 100–150 trials (Fig 1C) and our approach is consistent among individuals in a manner that is independent of the behavioral rig [20–23] (Fig 1D and 1E). Specifically, the sensitivity to α-pinene and eucalyptol, across different cohorts of mice that were tested in different behavioral chambers with different olfactometers, were similar (α-pinene: *p* = 0.824, *F* = 0.050; eucalyptol: *p* = 0.950, *F* = 0.004, sum of squares F-test) (Fig 1D and 1E).

**Fig 2:**
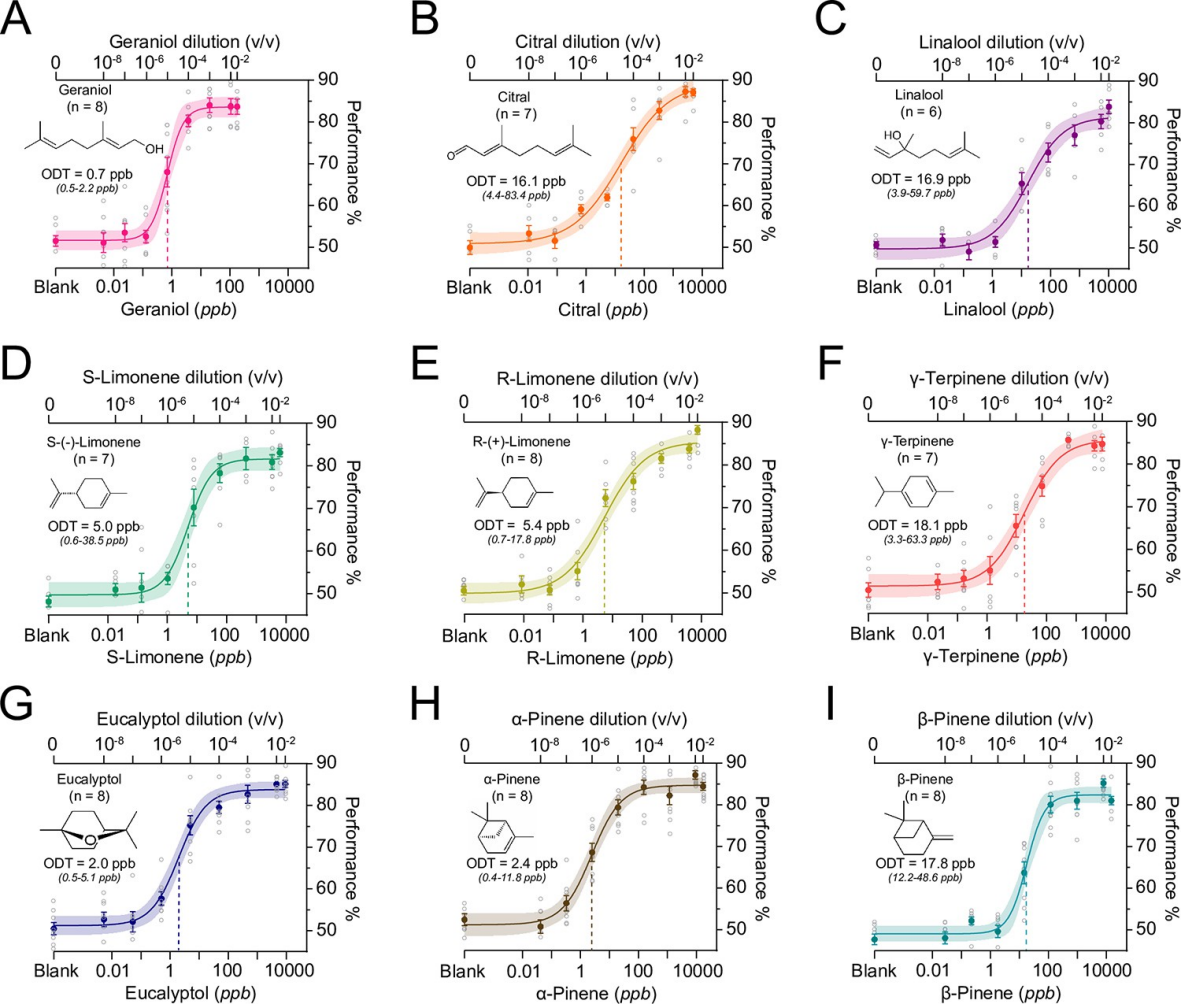
C57BL/6J mice are sensitive to monoterpenes. Psychometric curves to the acyclic monoterpenes: geraniol (**A**), citral (**B**), and linalool (**C**), the monocyclic monoterpenes: s-limonene (**D**), r-limonene (**E**), and γ-terpinene (**F**), and the bicyclic monoterpenes: eucalyptol (**G**), α-pinene (**H**), and β-pinene (**I**). Data were fitted using a Hill function. Maximal behavioral performance for each odor concentration is limited to ~85% (see methods). Behavioral threshold (ODT) is demarcated with a dashed line. Plots show mean +/- SE with shaded 95% confidence interval. Open circles denote individual behavioral performance at each concentration. Top X axis denotes the liquid dilution tested in the olfactometer.

**Fig 3:**
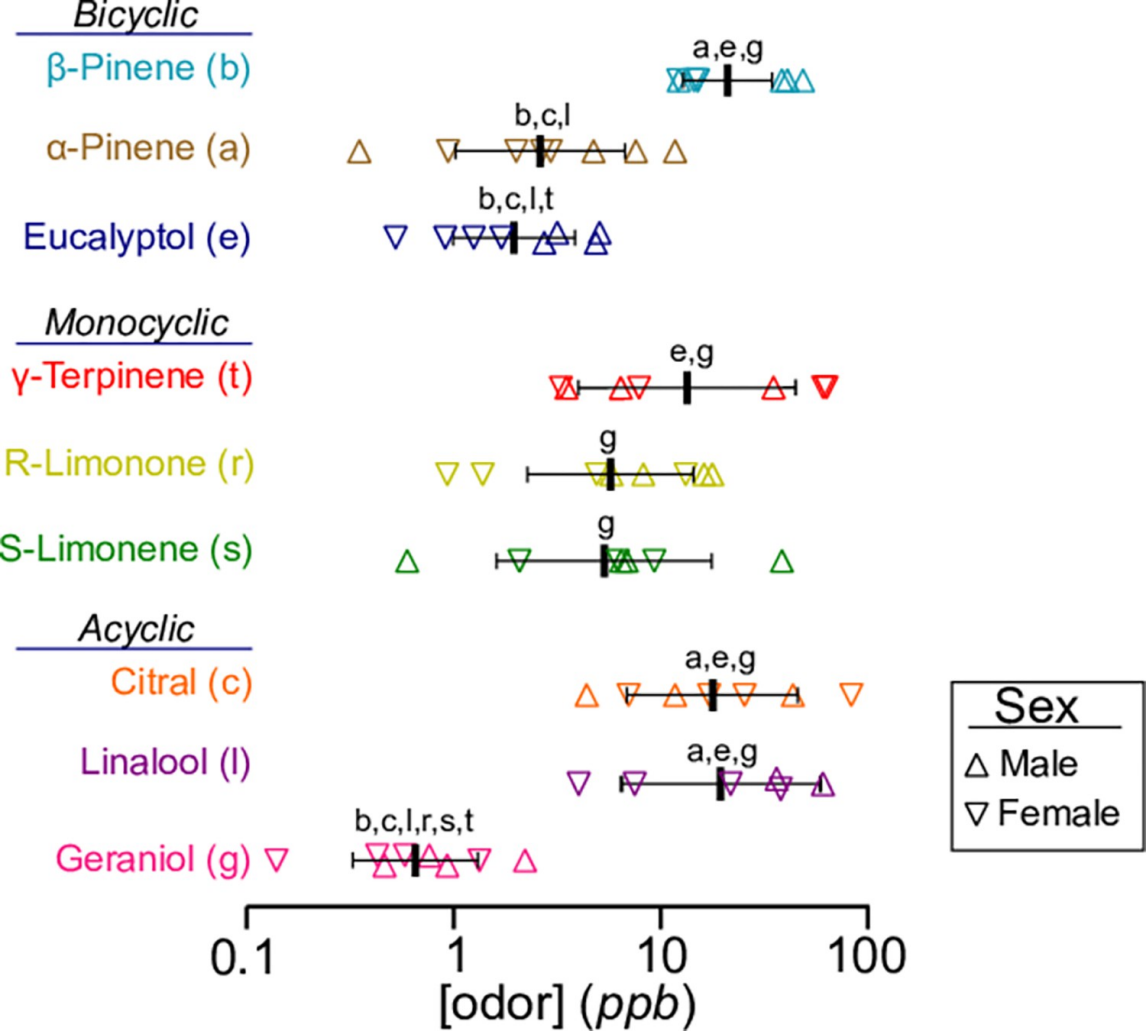
The sensitivity of individual mice to monoterpenes. Plots show mean+/- SD. Individual thresholds for each odorant are denoted with open triangles. Upward triangles denote males while downward triangles denote females. Letters signify statistical differences between two thresholds (p<0.05, two-way ANOVA with multiple comparisons). Statistical significance for these pairwise comparisons is displayed in both directions.

Mice differed in their sensitivity to these monoterpenes in a manner that was dependent on the sex of the animal (odor: *p* < 0.001, *F*(8, 49) = 12.39; sex: *p* = 0.004, *F*(1, 49) = 9.289; two-way ANOVA) (Figs 2 and 3). On average, females were more sensitive to monoterpenes than males (predicted log LS mean for males: -2.068 vs females: -2.380); however, a posthoc multiple comparison test did not yield any significant effects of sex within any single odor (geraniol p > 0.999; citral p > 0.999; linalool p > 0.999; r-limonene p = 0.231; s-limonene p = 0.518; γ-terpinene p > 0.999; eucalyptol p = 0.470; α-pinene p > 0.999; and β-pinene p > 0.999) (two-way ANOVA with multiple comparisons and Bonferroni correction). A power analysis determined that a group size of 32–48 animals (16–24 males and 16–24 females) per odor would be necessary to potentially observe statistical significance in odor sensitivity between sexes for specific odorants.

Of all the odorants tested, mice exhibited the greatest sensitivity to the acyclic monoterpene, geraniol. The average vapor-phase detection threshold to this odorant was 0.70 ppb (95% CI: 0.49–1.05 ppb), which is equivalent to a 1.02 x 10^−5^ v/v (95% CI: 0.62–1.78 x 10^−5^ v/v) dilution of geraniol in mineral oil (Figs 2A and 3). Individual mice differed in their sensitivity to this odorant by less than one order of magnitude (0.46–2.22 ppb). Mice were more sensitive to geraniol than other tested acyclic monoterpenes (citral *p* < 0.001; linalool *p* < 0.001), monocylic monoterpenes (γ-terpinene *p* < 0.001; r-limonene *p* = 0.008; s-limonene *p* = 0.002), and a single bicyclic monoterpene (β-pinene *p* < 0.001) (DF = 49; two-way ANOVA with multiple comparisons and Bonferroni correction) (Fig 3).

The average vapor-phase detection threshold for citral was 16.08 ppb (95% CI: 7.60–42.51 ppb), which is equivalent to a 4.51 x 10^−5^ v/v (95% CI: 2.18–11.71 x 10^−5^ v/v) dilution in mineral oil (Figs 2B and 3). Individual mice differed in their sensitivity to this odorant by more than one order of magnitude (4.39–83.37 ppb). Mice were less sensitive to citral than geraniol (*p* < 0.001) and two bicyclic monoterpenes (α-pinene *p* = 0.014 and eucalyptol *p* = 0.002) (DF = 49; two-way ANOVA with multiple comparisons and Bonferroni correction) (Fig 3).

The average vapor-phase detection threshold for linalool was 16.94 ppb (95% CI: 8.09–44.10 ppb), which is equivalent to a 1.74 x 10^−5^ v/v (95% CI: 0.77–4.98 x 10^−5^ v/v) liquid dilution in mineral oil (Figs 2C and 3). Individual mice differed in their sensitivity to this odorant by more than one order of magnitude (3.94–59.66 ppb). Mice were less sensitive to linalool than geraniol (*p*<0.001), two bicyclic monoterpenes (α-pinene *p* = 0.005 and eucalyptol *p* = 0.001) (DF = 49; two-way ANOVA with multiple comparisons and Bonferroni correction) (Fig 3).

Of the monocyclic monoterpenes tested, mice were most sensitive to s-limonene. The average vapor-phase detection threshold to this odorant was 5.01 ppb (95% CI: 2.78–9.13 ppb), which is equivalent to a 5.40 x 10^−6^ v/v (95% CI: 2.63–10.99 x 10^−6^ v/v) dilution of s-limonene in mineral oil (Figs 2D and 3). Individual mice differed in their sensitivity to this odorant by more than one order of magnitude (0.60–38.5 ppb). Mice were only less sensitive to s-limonene as compared to geraniol (*p* = 0.002; DF = 49; two-way ANOVA with multiple comparisons and Bonferroni correction) (Fig 3).

Mice displayed a similar sensitivity to its enantiomer, r-limonene. The average vapor-phase detection threshold to this odorant was 5.44 ppb (95% CI: 2.89–11.63 ppb), which is equivalent to a 9.17 x 10^−6^ v/v (95% CI: 4.68–20.58 x 10^−6^ v/v) dilution of r-limonene in mineral oil (Figs 2E and 3). Individual mice differed in their sensitivity to this odorant by more than one order of magnitude (0.69–17.79 ppb). Mice were only less sensitive to r-limonene as compared to geraniol (*p* = 0.008; DF = 49; two-way ANOVA with multiple comparisons and Bonferroni correction) (Fig 3).

Among the monocyclic monoterpenes tested, mice were least sensitive to γ-terpinene. The average vapor-phase detection threshold to this odorant was 18.10 ppb (95% CI: 9.18–36.73 ppb), which is equivalent to a 2.11 x 10^−5^ v/v (95% CI: 0.97–4.71 x 10^−5^ v/v) dilution of γ-terpinene in mineral oil (Figs 2F and 3). Individual mice differed in their sensitivity to this odorant by more than one order of magnitude (3.30–63.32 ppb). Mice were less sensitive to γ-terpinene as compared to the geraniol (*p* < 0.0001) and eucalyptol (p = 0.024) (DF = 49; two-way ANOVA with multiple comparisons and Bonferroni correction) (Fig 3).

Among the bicyclic monoterpenes tested, mice were most sensitive to eucalyptol. The average vapor-phase detection threshold to this odorant was 1.97 ppb (95% CI: 1.20–3.34 ppb), which is equivalent to a 3.97 x 10^−6^ v/v (95% CI: 2.40–6.75 x 10^−6^ v/v) dilution of eucalyptol in mineral oil (Figs 2G and 3). Individual mice differed in their sensitivity to this odorant by less than one order of magnitude (0.52–5.07 ppb). Mice were more sensitive to eucalyptol than β-pinene (*p* = 0.0003), γ-terpinene (*p* = 0.014), and citral (*p* = 0.002) (DF = 49; two-way ANOVA with multiple comparisons and Bonferroni correction) (Fig 3).

The average vapor-phase detection threshold to α-pinene was 2.45 ppb (95% CI: 1.49–4.05 ppb), which is equivalent to an 8.87 x 10^−7^ v/v (95% CI: 5.01–15.51 x 10^−7^ v/v) dilution in mineral oil (Figs 2H and 3). Individual mice differed in their sensitivity to this odorant by more than one order of magnitude (0.35–11.78 ppb). Mice were more sensitive to α-pinene than its isomer β-pinene (*p* = 0.002), and the acyclic monoterpenes citral (p = 0.014) and linalool (p = 0.005) (DF = 49; two-way ANOVA with multiple comparisons and Bonferroni correction) (Fig 3).

Among the bicyclic monoterpenes tested, mice were least sensitive to β-pinene. The average vapor-phase detection threshold to this odorant was 17.80 ppb (95% CI: 12.95–25.82 ppb), which is equivalent to a 1.23 x 10^−5^ v/v (95% CI: 0.87–1.86 x 10^−5^ v/v) dilution of β-pinene in mineral oil (Figs 2I and 3). Individual mice differed in their sensitivity to this odorant by less than one order of magnitude (12.18–48.62 ppb). Mice were less sensitive to β-pinene than its isomer α-pinene (*p* = 0.002), as well as eucalyptol (*p* = 0.0003) and geraniol (p < 0.0001) (DF = 49; two-way ANOVA with multiple comparisons and Bonferroni correction) (Fig 3).

**Fig 4:**
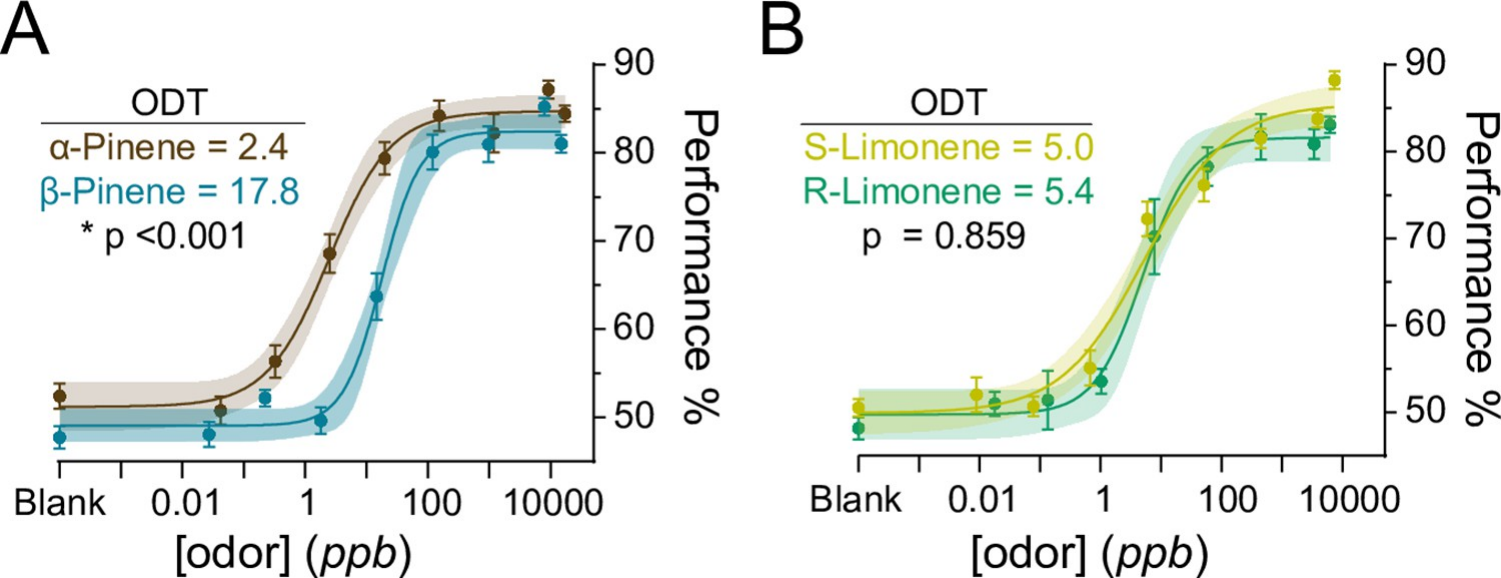
C57BL/6J mice are differentially sensitive to pinene isomers but not limonene enantiomers. Psychometric curves comparing α-pinene and β-pinene (**A**), as well as r-limonene and s-limonene (**B**). Data were fitted using a hill function. Maximal behavioral performance for each odor concentration is limited to ~85% (see methods). Plots show mean +/- SE with shaded 95% confidence interval. Statistical significance was calculated using a sum of squares F-test.

We also compared the average behavioral detection thresholds for the bicyclic monoterpene isomers: α-pinene and β-pinene, and the monocyclic monoterpene enantiomers: r-limonene and s-limonene. As stated above, mice were statistically more sensitive to α-pinene than β-pinene (*p* < 0.001, F = 38.55, sum of squares F-test; Fig 4A). In contrast, mice display similar sensitivities to r-limonene and s-limonene (*p* = 0.8599, F = 0.0313, sum of squares F-test; Fig 4B).

We did not find a statistically significant correlation between our monoterpene detection thresholds and either the atmospheric lifetime (r = 0.0418; *p* = 0.9221, Spearman correlation), volatility (r = -0.2594; *p* = 0.4980, Spearman correlation), or the air/mucus partition coefficient (r = 0.0084; *p* = 0.9915, Spearman correlation) of the odorants.

**Fig 5:**
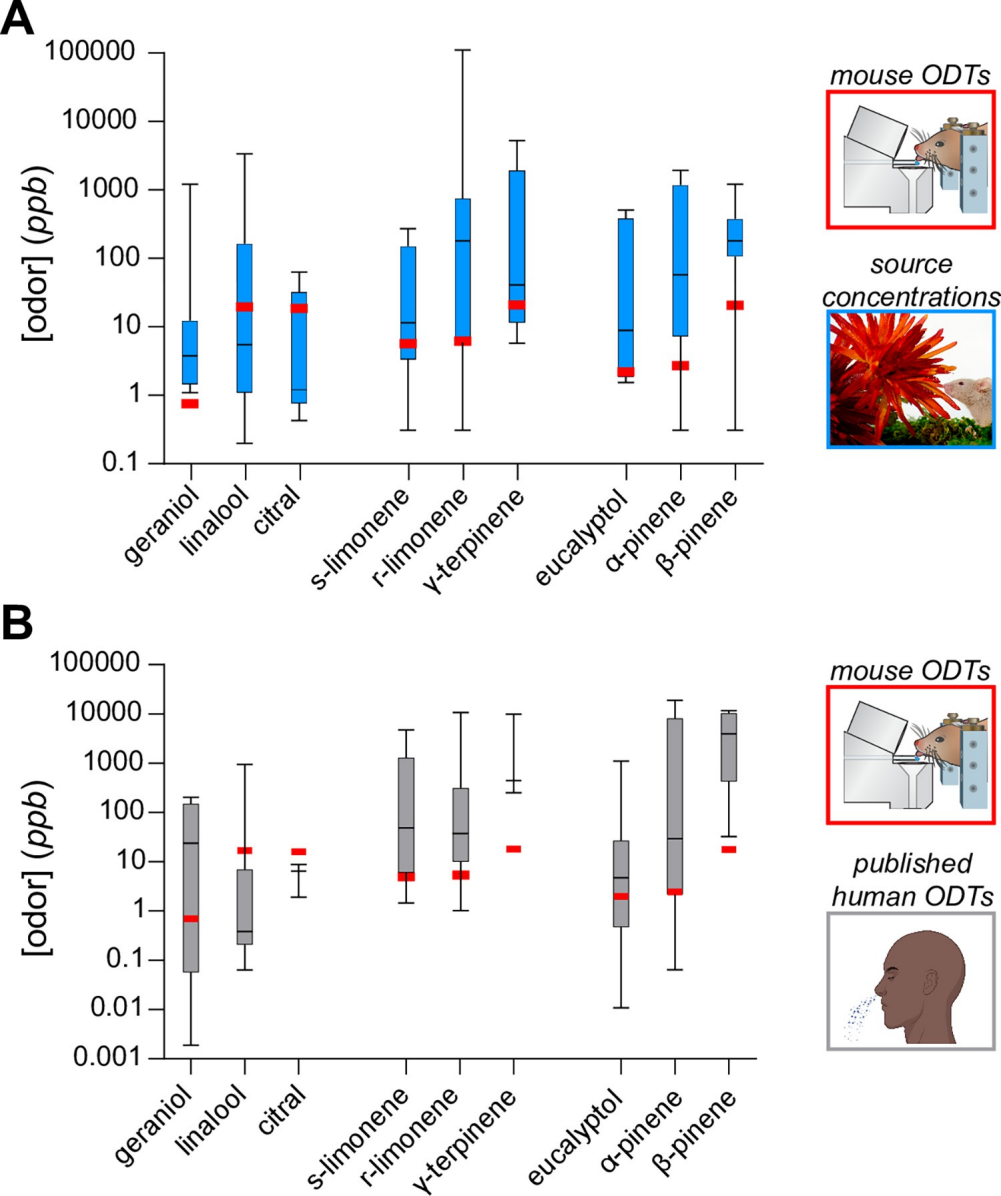
Contextualizing our estimations of monoterpene sensitivity in mice using published source concentrations and human detection thresholds. (**A**) Box and whisker plot of monoterpene concentrations emitted by sources relevant to mice, including various fruits, vegetables, nuts, and plants (see methods). All concentrations were determined using headspace volatile analysis via gas chromatography/mass spectrometry (GC/MS) or proton transfer reaction mass spectrometry (PTR-MS). (**B**) Box and whisker plot of human detection thresholds to monoterpenes compiled by Gemert, 2011. Red bars signify our estimation of the murine detection threshold for each odorant. Published human ODT icon was created with Biorender.com.

To estimate the maximal concentrations of monoterpenes naturally experienced by mice, we pooled data from studies analyzing the headspace concentration of monoterpenes emitted by various sources, including fruits, vegetables, nuts, and plants. For the majority of sources tested, monoterpene source concentrations ranged from ~1–1000 ppb, with a maximum of ~80 ppm (Fig 5A). In most cases, these source concentrations exceeded our measures of mouse sensitivity, indicating that mice can detect most (but not all) of the monoterpene volatiles emitted from these sources.

Lastly, to provide further context to our estimations of monoterpene sensitivity, we have plotted all the human thresholds for these odorants compiled by Gemert, 2011 [37] (Fig 5B). Our estimations of murine sensitivity for β-pinene and γ-terpinene are lower than all the compiled human thresholds. For other monoterpenes, including geraniol, limonene, eucalyptol, and α-pinene, our estimations of threshold in mice are lower than most, but not all, of the compiled human thresholds. In contrast, humans appear to be more sensitive to citral and potentially linalool. Despite the large discrepancies in estimations of human thresholds across studies, we find that mice have roughly similar sensitivities to these odorants as humans.

## Discussion

We found that C57BL/6J mice can reliably detect monoterpene concentrations in the parts per billion range. On average, mice are most sensitive to geraniol and least sensitive to γ-terpinene. We did not observe any statistically significant relationship between monoterpene sensitivity and either volatility, air/mucous coefficient, or the atmospheric lifetime of an odorant. We found that mice were more sensitive to α-pinene than its isomer, β-pinene, but exhibited similar thresholds to the limonene enantiomers. These robust estimations of monoterpene sensitivity define the minimum concentrations that should be utilized in functional studies and provide context for the vapor-phase delivery of these chemicals in studies investigating their biological activity in mice.

Our estimates of monoterpene sensitivity represent the first behavioral threshold measurements for six out of the nine odorants in mice. A previous study examining the contribution of NKCC1 found that wild-type mice exhibited a eucalyptol threshold of 242 ppb [19] (once we corrected the data for solvent effects using published equilibrium equations [35]). In contrast, we found that mice were approximately 100-fold more sensitive to this odorant, exhibiting a threshold of 2 ppb. As we have noted previously [22, 23], measurements of olfactory sensitivity can be influenced by a number of experimental factors including mouse strain, the definition of threshold, whether the animal is head-fixed or freely-moving, the method of odor delivery, and the training/testing procedure [60–63]. Please see Williams and Dewan (2020) [22], for a detailed discussion of our approach and how it compares to previous methods.

The sensitivity of mice to r-limonene and s-limonene has also been examined, but published thresholds dramatically differ. Blount and Coppola (2020) reported a solvent-corrected r-limonene threshold of 6.2 x 10^−14^ ppb (or 6.2 parts per septillion) in CD-1 mice [18]. In contrast, Joshi et al., (2006) tested the same strain of mice and found an average r-limonene threshold that is approximately 15 orders of magnitude higher (~117 ppb, once we corrected the data for solvent effects) [17]. Our estimation of the r-limonene sensitivity in C57BL/6J mice was more similar to the second study, yielding a threshold of 5.4 ppb. Further, Joshi et al., (2006) reported an average s-limonene threshold of 2.1 ppb (once corrected for solvent effects) [17], which was similar to our sensitivity measurement in C57BL/6J mice of 5.0 ppb. It should be noted that our mice are also highly trained and experience a very high degree of task replication, similar to the Blount and Coppola study [18], but exhibit more moderate thresholds. Thus, we would recommend that the minimum monoterpene concentrations utilized for functional studies, in mice, should be in the ppb range.

Monoterpene enantiomers are commonly used to probe the behavioral limits of odor discrimination [17, 18, 64–68], but have only been rarely examined in the context of sensitivity. As mentioned above, a previous study found CD-1 mice were more sensitive to s-limonene than r-limonene [17]. Although our estimation of s-limonene sensitivity in C57Bl/6J mice was similar to this previous study (2.1 vs 5.0 ppb), we did not observe any statistical difference in sensitivity to its enantiomer, r-limonene (5.4 ppb). It is possible that this discrepancy relates to strain differences in the receptor repertoire, but further research is needed. It is interesting to note that humans also exhibit similar thresholds for these enantiomers [69]. However, humans, rats, and mice [17, 18, 64, 70] can easily discriminate between these odorants, highlighting that despite similar sensitivities, their respective neural response is unique at least for the concentrations tested.

In addition to the main olfactory system, the trigeminal system can detect airborne chemicals and thus has the potential to impact sensitivity [71]. While the contribution of the trigeminal system to monoterpene detection has not been investigated in mice, a study utilizing anosmic humans found that geraniol, α-pinene, β-pinene, and both limonene enantiomers did not elicit any nasal pungency, while trigeminal sensitivity to citral and linalool was greater than three orders of magnitude higher than olfactory sensitivity [69]. Thus, it seems unlikely that mice are using their trigeminal system to enhance their sensitivity to monoterpenes, but further research is needed.

Our olfactory threshold measurements and published source concentrations can provide a guideline for experimenters to choose appropriate stimulus concentrations for functional studies. Based on our results, we would suggest a lower limit of ~20 ppb, as this concentration is above the detection threshold for all tested monoterpenes. It is important to note that one cannot infer this lower limit based solely on source concentrations, as the levels of specific odorants emitted by a natural source can be below an animal’s detection threshold. In fact, Dunkel et al., 2014 concluded that as few as 3% of the volatile chemicals emitted from a food source contribute to its specific smell [72]. Published source concentrations can be used to estimate the maximal stimulus concentration that a mouse could encounter in nature and thus, theoretically set the upper limit for relevant stimulus intensities. However, one clear caveat to our dataset is the limited number of studies that examine odorous sources within the natural environment of a mouse. Despite this shortcoming, we suggest an upper limit of ~1000 ppb for these monoterpenes. In conclusion, we would suggest utilizing vapor-phase monoterpene concentrations within the range of 20 to 1000 ppb for functional studies in mice.

Perceptual measures of sensitivity provide a mechanism to compare the olfactory system of different species. In fact, there is significant interest in how the olfactory capabilities of humans compares to a macrosmatic species like mice [73–77]. We found that the range of published human thresholds, albeit from a single collated source [37], were relatively similar to our sensitivity measures in mice. However, it is important to note that our estimations of murine sensitivity frequently fell below many of the median published human thresholds and mice appear to be more sensitive to both γ-terpinene and β-pinene. Despite these differences, the overall similarity between human and mouse sensitivity may indicate that experimenters may be able to use their own olfactory system to assess the quality of their odor delivery system, including testing for monoterpene contamination.

**Fig 6:**
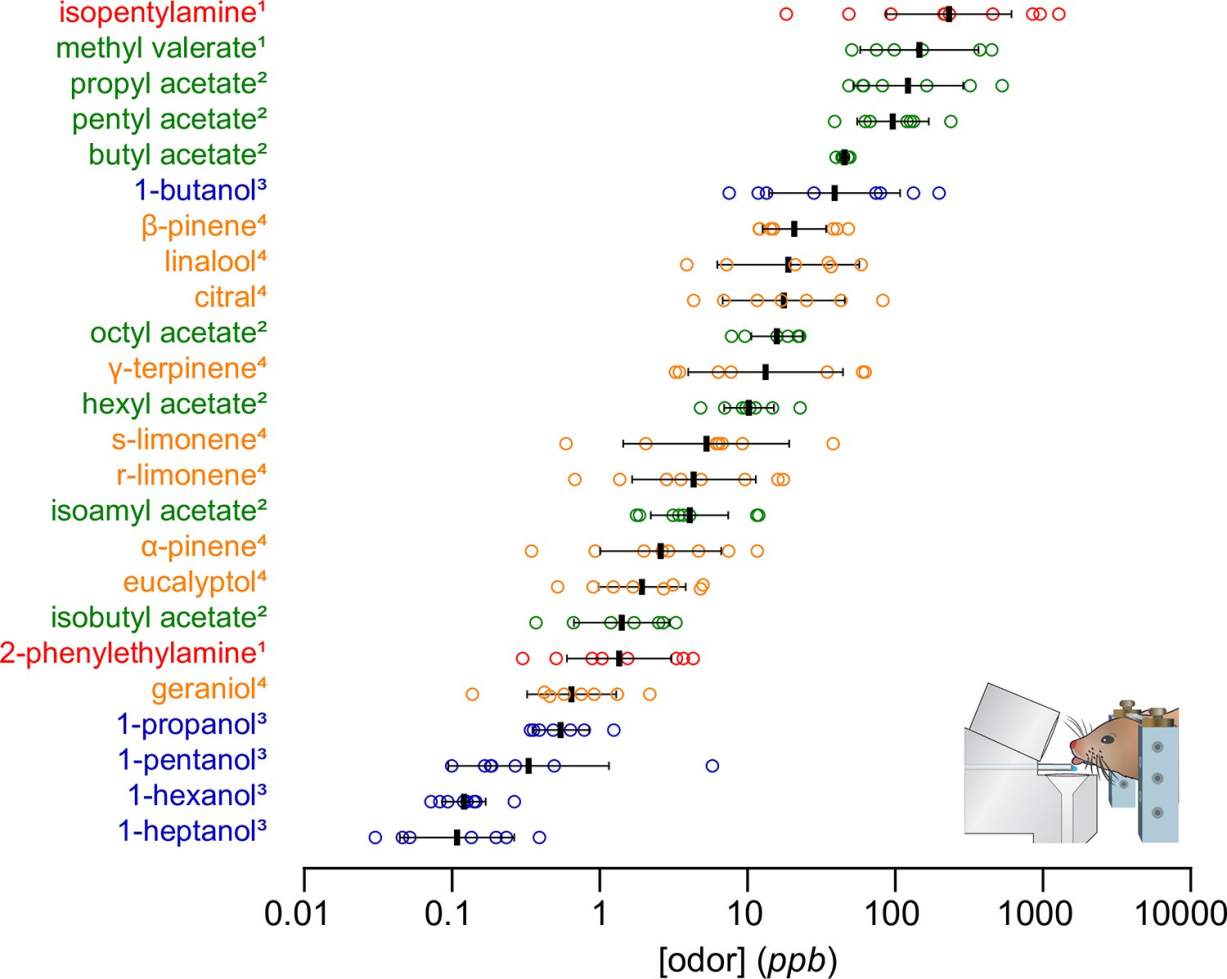
Summary of olfactory detection thresholds measured with the same approach. Plots show mean +/- SD. Individual thresholds for each odorant are denoted with open circles with the color denoting the chemical class (red–amine; green–ester, orange–monoterpene, blue–alcohol). Detection thresholds were corrected for solvent effects and obtained from 1—Dewan et al., (2018); 2- Jennings et al., (2022); 3- Williams and Dewan (2020); 4-current study.

Using the same method, we have previously measured the sensitivity of mice to aliphatic alcohols [22], esters [20, 21, 23], and amines [21]. We find that in general, mice are more sensitive to alcohols than these other odorants (Fig 6). Alcohols, carbonyls, and hydrocarbons have been identified as key volatiles produced by grain spoilage fungi [78]. The enhanced sensitivity of mice to alcohols could potentially assist in the identification of spoiled grains, which is a major food source for both wild and laboratory mice [79]. Likewise, the enhanced sensitivity to 2-phenylethylamine likely relates to its putative role as a predator odor [80, 81]. The high sensitivity to various monoterpenes, including geraniol, eucalyptol, and α-pinene was surprising, as these odorants have no known social relevance. Monoterpenes are among several classes of chemicals that are produced by plants for the purpose of chemical communication including attracting pollinators [82] and signaling damage from herbivores [83]. It is therefore possible that mice are eavesdropping on these volatile cues to detect potential food sources for their omnivorous diet, such herbivorous arthropods [84]. Interestingly, monoterpenes have also been found to have medicinal effects in both humans and mice [2, 5–12]; however, to our knowledge, there is no evidence of mice actively seeking natural sources of these chemicals.

## Conclusions

We have provided robust measurements of sensitivity in C57BL/6J mice to a series of acyclic, monocyclic, and bicyclic monoterpenes. These estimates serve to set the lower limit of monoterpene concentrations that should be used in functional studies. In addition, we have attempted to define an upper limit for these stimulus intensities by collating published measurements of monoterpene concentrations emitted from natural sources. Based on these two complementary datasets, we would suggest utilizing vapor-phase concentrations of these monoterpenes within the range of 20 to 1000 ppb for functional studies in mice. It is our hope that these analyses will help researchers use appropriate concentrations for functional studies employing monoterpenes and provide context for the vapor-phase delivery of these odorants to investigate their biological activity in rodent models.

## Supporting information

S1 Fig Photoionization device (PID) traces of 250 sequential stimulus presentations from a single vial of the acyclic monoterpenes: geraniol (**A**), citral (**B**), and linalool (**C**), the monocyclic monoterpenes: s-limonene (**D**), r-limonene (**E**), and γ-terpinene (**F**), and the bicyclic monoterpenes: eucalyptol (**G**), α-pinene (**H**), and β-pinene (**I**). Shaded area signifies 2 second stimulus period.
(TIF)
