# Correction: The behavioral sensitivity of mice to acyclic, monocyclic, and bicyclic monoterpenes

**DOI:** 10.1371/journal.pone.0301717

**Published:** 2024-03-29

**Authors:** The PLOS ONE Staff

## Notice of republication

This article was republished on March 21, 2024, to correct an error in the PDF version. The figures were erroneously missing from the PDF. The publisher apologizes for the error. Please download this article again to view the corrected version. The originally published, uncorrected article and the republished, corrected articles are provided here for reference.

## Supporting information

S1 File Originally published, uncorrected article. (PDF)

S2 File Republished, corrected article. (PDF)

## References

[1] Williams E, Pauley A, Dewan A (2024) The behavioral sensitivity of mice to acyclic, monocyclic, and bicyclic monoterpenes. PLoS ONE 19(2): e0298448. doi: 10.1371/journal.pone.0298448 38394306 PMC10890753

