# Supplementary figures and images for: The behavioral sensitivity of mice to acyclic, monocyclic, and bicyclic monoterpenes
Source: PLoS One. 2024 Feb 23;19(2):e0298448. doi: 10.1371/journal.pone.0298448 (PMC10890753; doi:10.1371/journal.pone.0298448)

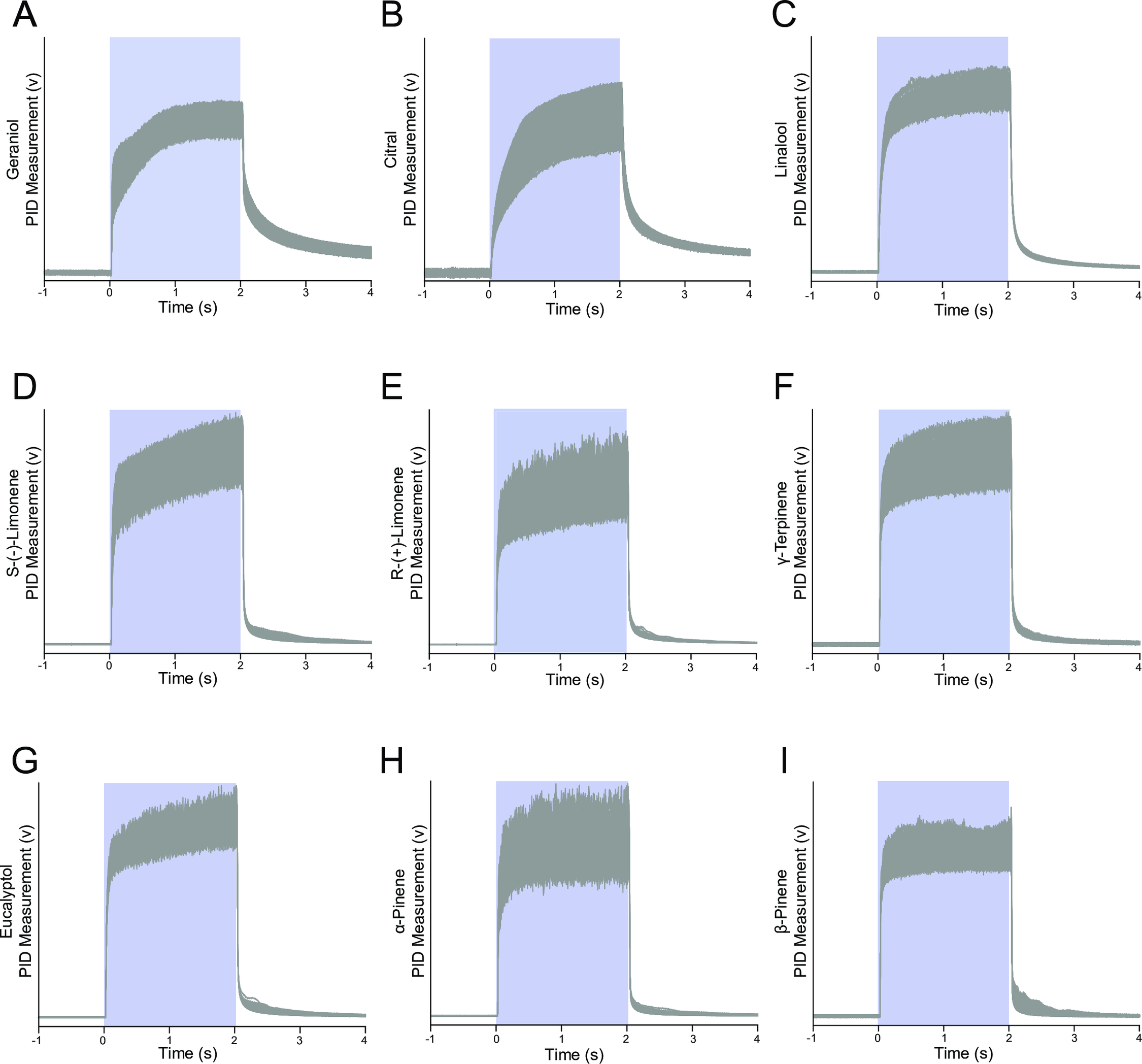

Supplement: S1 Fig — Photoionization device (PID) traces of 250 sequential stimulus presentations from a single vial of the acyclic monoterpenes: geraniol (A), citral (B), and linalool (C), the monocyclic monoterpenes: s-limonene (D), r-limonene (E), and γ-terpinene (F), and the bicyclic monoterpenes: eucalyptol (G), α-pinene (H), and β-pinene (I). Shaded area signifies 2 second stimulus period. (TIF) [file pone.0298448.s001.tif]
