# Supplementary material for: Correction: The behavioral sensitivity of mice to acyclic, monocyclic, and bicyclic monoterpenes
Source: PLoS One. 2024 Mar 29;19(3):e0301717. doi: 10.1371/journal.pone.0301717 (PMC10980186; doi:10.1371/journal.pone.0301717)
Supplement: S2 File — (PDF) [file pone.0301717.s002.pdf]

PLOS ONE

RESEARCH ARTICLE

The behavioral sensitivity of mice to acyclic, monocyclic, and bicyclic monoterpenes

Ellie Williams, Austin Pauley, Adam Dewan*

Department of Psychology, Program in Neuroscience, Florida State University, Tallahassee, FL, United States of America

* dewan@psy.fsu.edu

Check for updates

OPEN ACCESS

Citation: Williams E, Pauley A, Dewan A (2024) The behavioral sensitivity of mice to acyclic, monocyclic, and bicyclic monoterpenes. PLoS ONE 19(2): e0298448. https://doi.org/10.1371/journal.pone.0298448

Editor: Jorddy Neves Cruz, Universidade Federal do Para, BRAZIL

Received: December 1, 2023

Accepted: January 23, 2024

Published: February 23, 2024

Copyright: © 2024 Williams et al. This is an open access article distributed under the terms of the Creative Commons Attribution License, which permits unrestricted use, distribution, and reproduction in any medium, provided the original author and source are credited.

Data Availability Statement: The data that support the findings of this study are available for download at https://github.com/OlfactoryBehaviorLab.

Funding: This work was supported by the National Institutes of Health under grant [2T32DC000044] awarded to fund EW, and the Whitehall Foundation Research Grant [2020-05-02] awarded to AD. The funders had no role in study design, data collection and analysis, decision to publish, or preparation of the manuscript.

Competing interests: The authors have declared that no competing interests exist.

Abstract

Monoterpenes are a large class of naturally occurring fragrant molecules. These chemicals are commonly used in olfactory studies to survey neural activity and probe the behavioral limits of odor discrimination. Monoterpenes (typically in the form of essential oils) have been used for centuries for therapeutic purposes and have pivotal roles in various biological and medical applications. Despite their importance for multiple lines of research using rodent models and the role of the olfactory system in detecting these volatile chemicals, the murine sensitivity to monoterpenes remains mostly unexplored. We assayed the ability of C57BL/6J mice to detect nine different monoterpenes (the acyclic monoterpenes: geraniol, citral, and linalool; the monocyclic monoterpenes: r-limonene, s-limonene, and γ-terpinene; and the bicyclic monoterpenes: eucalyptol, α-pinene, and β-pinene) using a head-fixed Go / No-Go operant conditioning assay. We found that mice can reliably detect monoterpene concentrations in the low parts per billion (*ppb*) range. Specifically, mice were most sensitive to geraniol (threshold: 0.7 ppb) and least sensitive to γ-terpinene (threshold: 18.1 ppb). These estimations of sensitivity serve to set the lower limit of relevant monoterpene concentrations for functional experiments in mice. To define an upper limit, we estimated the maximum concentrations that a mouse may experience in nature by collating published headspace analyses of monoterpene concentrations emitted from natural sources. We found that natural monoterpenes concentrations typically ranged from ~1 to 1000 ppb. It is our hope that this dataset will help researchers use appropriate monoterpene concentrations for functional studies and provide context for the vapor-phase delivery of these chemicals in studies investigating their biological activity in mice.

Introduction

Monoterpenes are a large class of volatile metabolites produced by plants in order to attract pollinators, reduce the impact of biotic / abiotic stressors, and even trigger an immune response in distant plants to combat herbivory [1, 2]. Their low molecular weight and high lipophilicity confer high absorption and membrane penetration capacities contributing to their biological action and medicinal potential [3, 4]. Specifically, monoterpenes (and their derivatives) have been shown to have analgesic, antioxidant, and anti-inflammatory properties

in humans [2, 5] as well as antioxidant, sedative, antinociceptive, and anxiolytic-like effects in mice [6–12], and have been widely studied for their potential as natural insecticides [13, 14]. Monoterpenes may also act as signaling molecules—influencing energy metabolism with anti-diabetic and anti-obesity properties [15]. Beyond these medicinal applications, monoterpenes are also prominent components of the flavor of many fruits and vegetables [16] and thus contribute to ingestive behavior. Despite the clear importance of monoterpenes for multiple lines of research using rodent models and the role of the olfactory system in detecting these volatile chemicals, the murine sensitivity to monoterpenes remains mostly unexplored.

The goal of this study was to define the detection threshold of mice to volatile odorants within each monoterpene division, specifically geraniol, citral, and linalool (acyclic monoterpenes); r-limonene, s-limonene, and γ-terpinene (monocyclic monoterpenes); and eucalyptol, α-pinene, and β-pinene (bicyclic monoterpenes). Previous studies have estimated murine sensitivity for only three of these compounds (eucalyptol, r-limonene, and s-limonene) [17–19]. Even among this limited odor set, our understanding of murine sensitivity remains clouded, as the only chemical tested in multiple studies (r-limonene) has published thresholds that differ by ~15 orders of magnitude [17, 18]. Our established behavioral method has resulted in estimates of olfactory sensitivity that are internally consistent, across both individuals and different cohorts even when tested in different behavioral rigs by different experimenters [20–24]. Our results will complement these previous studies by providing the first estimation of sensitivity for six of these monoterpenes and help clarify the large discrepancy between published monoterpene thresholds in mice.

Monoterpenes are also commonly used in olfactory research to survey neural activity in a variety of olfactory regions, probe the behavioral limits of odor discrimination, and assess odor-structure activity relationships in mice [17, 25–32]. The stimulus intensities used in these studies vary widely as appropriate concentrations have yet to be defined. Our estimations of monoterpene sensitivity can help define the low-end of relevant monoterpene concentrations that should be used in functional experiments. To define the high-end of relevant concentrations, we have combed the literature for headspace analyses of monoterpenes emitted from natural sources. Together, these data will hopefully guide researchers in choosing appropriate stimulus concentrations for functional experiments utilizing monoterpenes.

Materials and methods

The methods utilized in this study are well documented [20–23], including a step-by-step guide to our surgical approach, behavioral training, odor delivery, and operant conditioning assay [24].

Animals

Male and female C57BL/6J mice (16 M; 16 F) were housed in same-sex cages until head-bar surgery. Mice (10–14 weeks old) were anesthetized with isoflurane at a dosage of 2–3% in oxygen, administered buprenorphine ER (0.1 mg/kg) as an analgesic, and lidocaine (2 mg/kg) as a local anesthetic. A custom head-bar was affixed to the skull using 2–3 micro-screws and secured using dental cement. After surgery, mice were individually housed and given at least three days to recover. Following recovery, mice were water restricted for at least two weeks before training in a water-rewarded conditioning paradigm [24]. At the conclusion of the experiment, mice were euthanized via carbon dioxide inhalation. All procedures conducted were reviewed and approved by the Florida State University Animal Care and Use Committee [Protocol # 202100020]. All efforts were made to ensure the health and welfare of the animal throughout the experiment.

Odor stimuli

A set of nine monoterpenes was used in this study, including three acyclic monoterpenes: linalool (CAS# 78-70-6), geraniol (CAS# 106-24-1), and citral (CAS# 141-27-5); three monocyclic monoterpenes: r-limonene (CAS# 5989-27-5), s-limonene (CAS# 5989-54-8), and γ-terpinene (CAS# 99-85-4); and three bicyclic monoterpenes: α-pinene (CAS# 80-56-8), β-pinene (CAS# 127-91-3), and eucalyptol (CAS# 470-82-6). The use of both isomer (α-pinene / β-pinene) and enantiomer (r-limonene / s-limonene) pairs allowed for a comparison of mouse sensitivity across structurally similar molecules. All odorants were obtained from Millipore-Sigma at the highest available purity (>98%), stored under nitrogen, and housed in a chemical storage cabinet. Odorants were diluted with mineral oil (CAS# 8042-47-5) within an odor-free chemical safety cabinet with the use of filtered pipette tips. The maximum odorant concentration tested was a 2:100 dilution (or 2% v/v) for all odorants.

To determine if behavioral sensitivity was correlated with specific odorant features, we obtained/calculated the air/mucus odorant partition coefficient, volatility, and atmospheric lifetime for each odorant. The air/mucus odorant partition coefficient is defined by the ratio of odorant concentration in the air phase to the concentration in the mucus at the air-mucus interface [33]. The air/mucus odorant partition coefficient (β) was calculated based on the following equation [33]:

$$\log\beta = \log\beta_{\mathrm{water}} - (\log P - 1) \times 0.524$$

where β_{water} is the air/water partition coefficient and P is the octanol/water partition coefficient, both of which were obtained through U.S. Environmental Protection Agency database [Estimation Program Interface [Epi] Suite tool; www.epa.gov/oppt/exposure/pubs/episuite.htm]. Atmospheric chemical lifetime and volatility measures for each odorant were also obtained through [Epi] Suite tool (EPA). Atmospheric lifetime is a measure of the rate of oxidation for an odorant and was previously correlated to human olfactory sensitivity [34].

The vapor-phase concentration for eight of the odorants, were determined according to published equilibrium equations [35]. The relationship between the liquid- and vapor-phase concentration of citral in mineral oil was determined using a photoionization detector (PID) based approach [36]. Briefly, we measured the net charge resulting from the ionization of the headspace above pure and diluted citral concentrations using a calibrated PID with the use of a correction factor. To calculate the correction factor for citral, a sealed bottle containing a small volume of this odorant (2.5μl and subsequently verified with 5.0 μl) was heated beyond its boiling point. With the use of three-way valves, a mass flow controller (MFC) directed the vaporized sample from the temporarily sealed bottle to the PID. The vapor concentration (within the bottle) was divided by the equivalent isobutylene concentration (as determined by the PID voltage) to calculate the correction factor for that odorant. This resulted in a correction factor of 2.05 and a saturated vapor concentration of 112 ppm for citral. This measurement closely matched the predicted vapor concentration of 120 ppm as calculated by the following equation:

$$C\,(ppm) = \frac{P_{vap}}{P_{atm}}\left(10^{6}\right)$$

Where C is the concentration in ppm, P_{vap} is the vapor pressure of citral (0.0193 mmHg), and P_{atm} is the atmospheric pressure in mmHg at 25˚C. To estimate the relationship between the liquid and vapor-phase concentration of citral, five PID measurements were taken from each liquid dilution with a 30 second inter-trial interval. The calibrated net charge for each trial was multiplied by the citral correction factor to determine the vapor-phase concentration resulting

from the associated liquid dilution. These data were plotted and fitted with a power function ($[C]_{vap} = a[C]_{liq}^{\beta}$; Prism Graphpad).

$$[\mathrm{citral}]_{\mathrm{vap}} = 26.3*[\mathrm{citral}]_{\mathrm{MO}}{}^{0.90}.$$

To estimate the solvent-corrected vapor-phase threshold of a published study [17] testing murine sensitivity to r-limonene and s-limonene in diethyl phthalate, we measured the correction factors and equilibrium equations associated with these odorants in diethyl phthalate using the same method as above. Our analysis yielded a correction factor of 0.72 for both odorants and the following equilibrium equations:

$$[\mathrm{s-limonene}]_{\mathrm{vap}} = 82.7*[\mathrm{s-limonene}]_{\mathrm{DEP}}{}^{0.92}$$

$$[\mathrm{r-limonene}]_{\mathrm{vap}} = 95.9*[\mathrm{r-limonene}]_{\mathrm{DEP}}{}^{0.90}$$

Our estimation of the saturated vapor concentration of these odorants (r-limonene: 1924 ppm and s-limonene: 1932 ppm) was very similar to the predicted vapor concentration of 1907 ppm.

Stimulus delivery

Disposable 40 mL amber glass vials filled with 15 mL of diluted odorant (or solvent) were attached to the 8-channel olfactometer (Fig 1A) and pressurized before the start of the first trial. Nitrogen gas regulated by a 100 mL/min MFC flowed through the selected vial before it was diluted 10 times by the main air flow stream (regulated by a 900 mL/min air MFC). During stimulus delivery, the final valve swapped the flow to the animal from clean air to diluted odorant. The selected vial is actuated 0.6-s before stimulus delivery to allow the odor concentration to reach equilibrium before delivery to the animal. The odor port is attached to a micromanipulator to standardize the distance to the nose for each head-fixed animal. To verify the stability and reproducibility of our odorant presentations, a PID was used in place of the mouse. A single vial containing 15 mL of the diluted odorant was repeatedly actuated (250 times) with a 15-s inter-trial interval (Fig 1B and S1 Fig).

Behavioral assay

Water-restricted mice were trained to report the detection of odor in a Go/No-Go task [20–24]. Each cohort of mice initially consisted of four males and four females (age-matched). Cohorts were tested on a maximum of three odorants to limit over-training and the probability that mice would learn to solve the task using non-odor cues (see below). Individual mice were excluded from the experiment if they failed to reach the training criterion for a particular odorant (n = 4 out of 32) or learned to solve the task using non-odor cues (n = 0 out of 32).

Behavioral training consisted of two stages. Stage 1—the mice were trained to receive a water reward if they licked during the stimulus period. Stage 2—mice were trained in a Go / No-Go odor detection task. Sessions typically lasted 120–300 trials and were terminated after the mice missed three Go trials in a row or the mouse reached 300 trials. Behavioral performance was determined by the number of correct responses (hits + correct rejections) divided by the total number of trials (after the initial Go trials). Upon reaching criterion (two sessions >90% correct), mice were subsequently tested in the thresholding assay. Mice that did not reach criterion in a maximum of 4 days were excluded.

To determine behavioral thresholds, mice were only tested on one concentration per day. This approach eliminated any masking/adaptation effects resulting from the contamination of

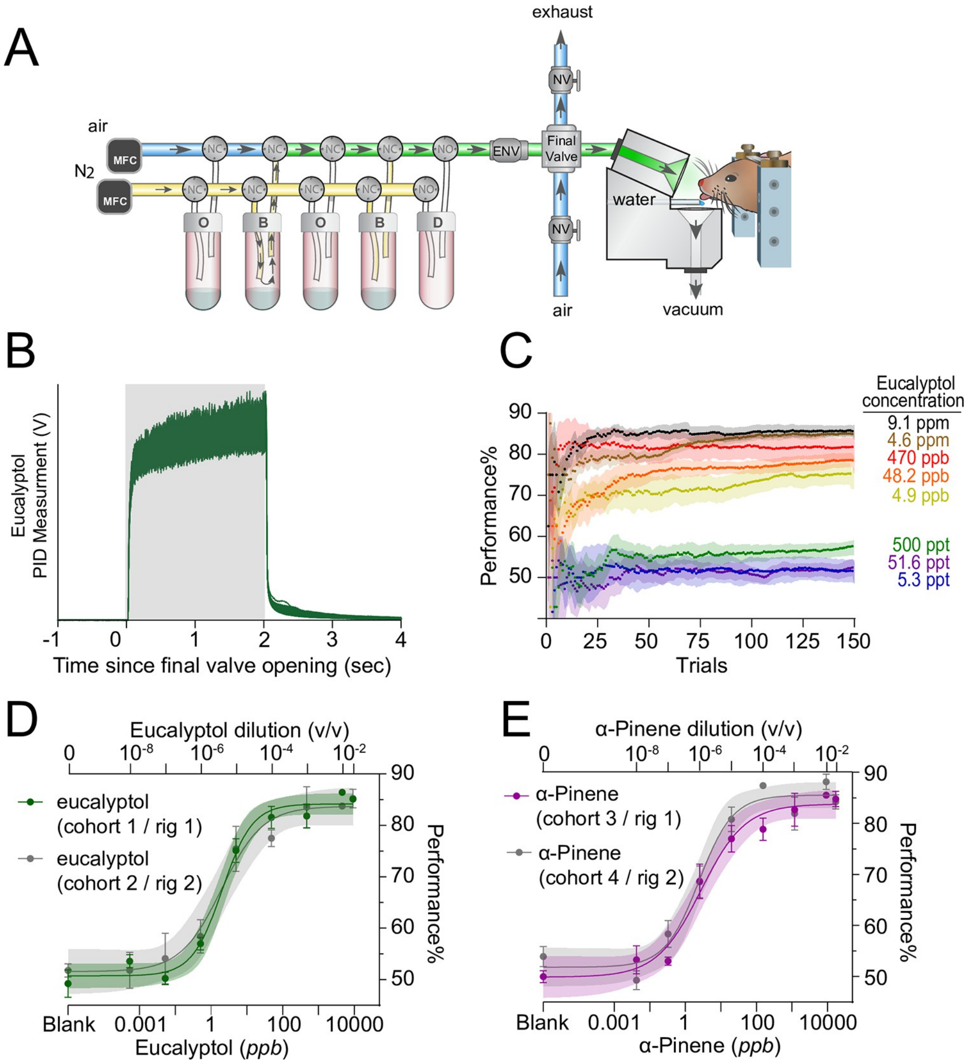

Fig 1. To measure behavioral detections thresholds, we used a head-fixed Go / No-Go operant conditioning assay. (**A**) Odorants were delivered using an 8-channel flow dilution olfactometer with mass flow controllers (MFC) that switch between a pressure-balanced dummy (D) vial (via normally open valves, NO) and either odor (O) or solvent-only (B) vials (via normally closed valves, NC). Odorized air is directed to exhaust to allow the stimulus to reach equilibrium prior to stimulus delivery. During stimulus application, a final valve (dual-synchronous solenoid valve) redirects pressure-balanced, odorized air from exhaust to the animal. Manual needle valves (NV) are used to pressure-balance the air and exhaust lines, while an electronic valve (ENV) is used to pressure-balance individual vials across the olfactometer manifold. At the conclusion of the trial, the final valve returns the pressure-balanced clean air to the animal. (**B**) Photoionization detector (PID) traces of 250 stimulus presentations of eucalyptol. Shaded area signifies two second stimulus period. (**C**) Average cumulative behavioral performance across 150 trials for all concentrations of eucalyptol. Initial go trials are not included. Line signifies mean with shaded SE. (**D-E**) Our experimental approach did not differ across mouse sub-cohorts (N = 4) or different behavioral setups / olfactometers. Data were fitted using a Hill function. Maximal behavioral performance for each odorant concentration is limited to ~85% (see methods). Plots show mean +/- SE with shaded 95% confidence interval.

https://doi.org/10.1371/journal.pone.0298448.g001

the olfactometer by higher concentrations of the target odor. The olfactometer was loaded with three solvent (Go) vials, three diluted odor (No-Go) vials, and a single solvent (No-Go) vial. Each vial was replaced daily, and their positions were randomized. The solvent No-Go vial (or "cheating check") served to test whether the mice were using cues other than the presence or absence of the target odor to maximize performance. This solvent No-Go vial should

be indistinguishable from other solvent-only Go vials unless the animal is using non-odor cues to maximize performance and the associated water reward. Thus, mice are "cheating" at this task if they are able to reject (i.e. not lick) the solvent No-Go vial at a frequency higher than the percentage of misses (i.e. not licking during a solvent Go vial). If this occurred, the session was excluded from the analysis. If this occurred multiple times (i.e., > 2) over the course of an experiment, the mouse was removed from the experimental group. Since this check is included in our thresholding analysis, the maximum performance a mouse can attain using only odor cues in this experiment is approximately 85% (in contrast to stage 2 training in which the mice can achieve 100% behavioral performance). After the completion of all odorant concentrations, the mouse's ability to discriminate between vials using non-odor cues was again tested by loading the olfactometer with only solvent vials. These data are included in each figure.

At the end of each day, the olfactometer (including the manifolds and all tubing) was flushed with acetone, 70% isopropanol, and nanopure water, and then was dried with pressurized clean air overnight. The vial caps and tubing were also cleaned with acetone, isopropanol, and nanopure water, and then dried overnight.

Statistical analysis

Behavioral performance for each odorant was fitted with a four-parameter Hill equation (Prism Graphpad).

$$R = R_{min} + C^{n} * \frac{R_{max} - R_{min}}{[C^{n} + C_{\S}^{n}]}$$

where R is the behavioral accuracy, C is odor concentration, $C_{½}$ is the concentration at half-maximal performance, and n is the Hill coefficient. The goodness-of-fit for odorant-specific Hill equation parameters were compared with a sum-of-squares F test (Prism Graphpad). Behavioral thresholds ($C_{½}$) were compared between odorants and sexes using a two-way ANOVA with multiple comparisons (Prism GraphPad).

Human thresholds and source concentrations

To contextualize our estimations of murine sensitivity, we collated published measures of human sensitivity and monoterpene concentrations emitted from natural sources. Estimations of human sensitivity were obtained from Gemert, 2011 [37] and consisted of 72 monoterpene thresholds from 37 sources. Estimations of natural source concentrations were taken from published studies utilizing either headspace volatile analysis via gas chromatography/mass spectrometry (GC/MS) or proton transfer reaction mass spectrometry (PTR-MS). This literature review resulted in data from 22 studies analyzing the headspace volatile concentrations of monoterpenes emitted from 37 compounds. Importantly, we limited our analysis to sources for which 1) mice could reasonably experience and 2) methods in which the sample was not modified by solvent extraction methods. Please note that the PTR-MS method is limited in its ability to unambiguously identify compounds with the same mass/charge ratio. In such cases where a volatile compound was identified as one of a couple possibilities (e.g., r-limonene or s-limonene), the concentration value was assigned to both odorants. Our literature review included headspace volatile analyses from several vegetables (carrot, tomato, and cucumber [38–41]), fruits / fruit juices (blueberry, peach, tangerine, tangerine peel, grapefruit, strawberry, strawberry sepals, orange juice, apple, quince, litchi, and loquat [40, 42–50]), spices (saffron, black and white pepper, cinnamon, and nutmeg [51]), and several other items (tea, almonds, cocoa beans, animal urine, bread, olive oil, truffles, and pine needles [40, 52–59]).

Results

To measure behavioral detection thresholds, we used a head-fixed Go / No-Go operant conditioning assay (Fig 1A). Our stimulus delivery system resulted in consistent odor pulses throughout a session (Fig 1B and S1 Fig). As shown previously, behavioral performance at each concentration can be predicted after 100–150 trials (Fig 1C) and our approach is consistent among individuals in a manner that is independent of the behavioral rig [20–23] (Fig 1D and 1E). Specifically, the sensitivity to α-pinene and eucalyptol, across different cohorts of mice that were tested in different behavioral chambers with different olfactometers, were similar (α-pinene: $p = 0.824$, $F = 0.050$; eucalyptol: $p = 0.950$, $F = 0.004$, sum of squares F-test) (Fig 1D and 1E).

Mice differed in their sensitivity to these monoterpenes in a manner that was dependent on the sex of the animal (odor: $p < 0.001$, $F(8, 49) = 12.39$; sex: $p = 0.004$, $F(1, 49) = 9.289$; two-way ANOVA) (Figs 2 and 3). On average, females were more sensitive to monoterpenes than males (predicted log LS mean for males: -2.068 vs females: -2.380); however, a posthoc multiple comparison test did not yield any significant effects of sex within any single odor (geraniol $p > 0.999$; citral $p > 0.999$; linalool $p > 0.999$; r-limonene $p = 0.231$; s-limonene $p = 0.518$; γ-terpinene $p > 0.999$; eucalyptol $p = 0.470$; α-pinene $p > 0.999$; and β-pinene $p > 0.999$) (two-

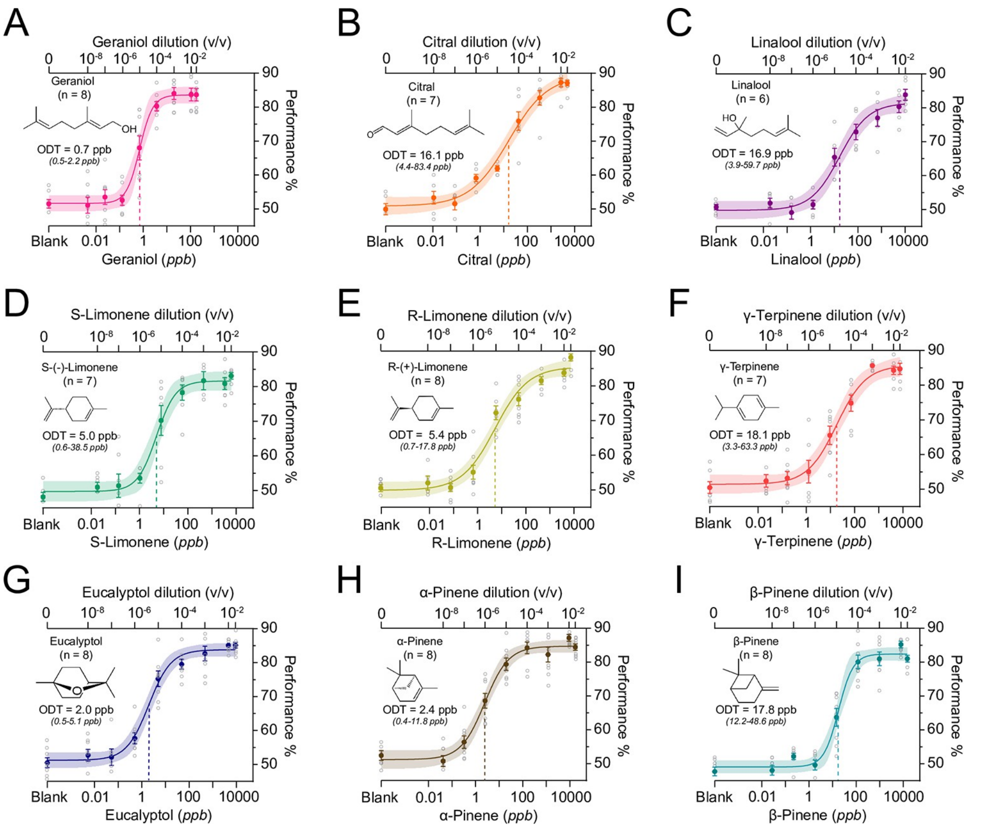

Fig 2. C57BL/6J mice are sensitive to monoterpenes. Psychometric curves to the acyclic monoterpenes: geraniol (**A**), citral (**B**), and linalool (**C**), the monocyclic monoterpenes: s-limonene (**D**), r-limonene (**E**), and γ-terpinene (**F**), and the bicyclic monoterpenes: eucalyptol (**G**), α-pinene (**H**), and β-pinene (**I**). Data were fitted using a Hill function. Maximal behavioral performance for each odor concentration is limited to ~85% (see methods). Behavioral threshold (ODT) is demarcated with a dashed line. Plots show mean +/- SE with shaded 95% confidence interval. Open circles denote individual behavioral performance at each concentration. Top X axis denotes the liquid dilution tested in the olfactometer.

https://doi.org/10.1371/journal.pone.0298448.g002

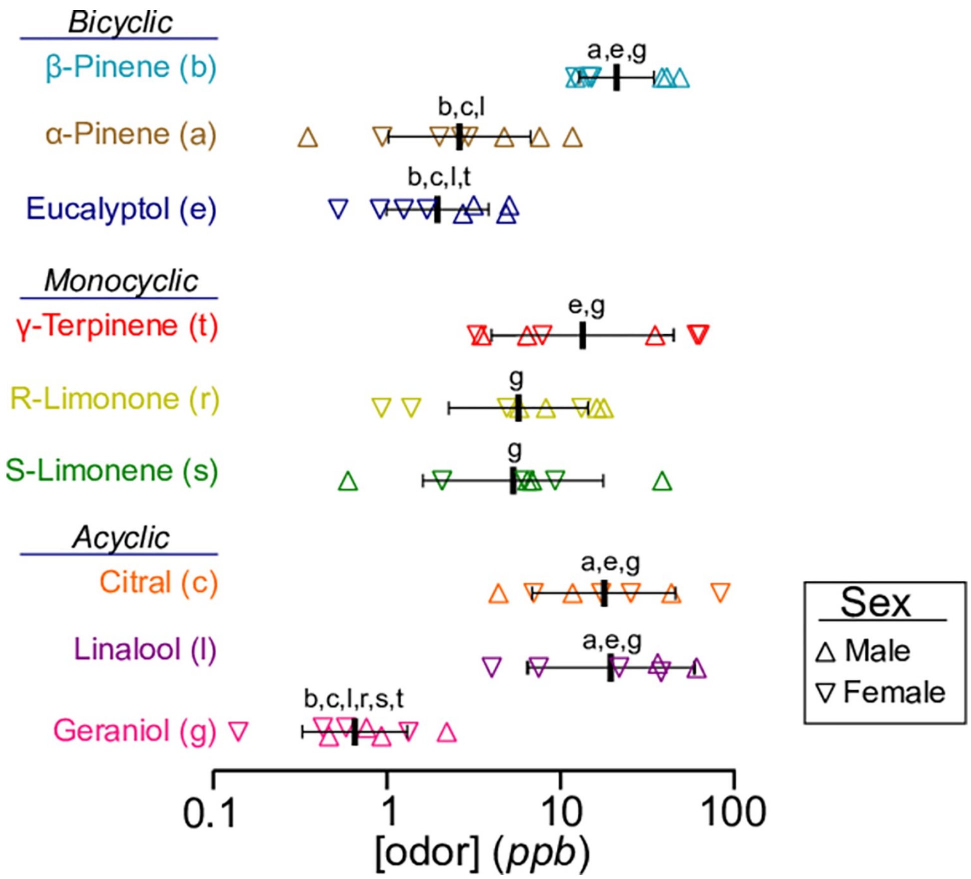

Fig 3. The sensitivity of individual mice to monoterpenes. Plots show mean+/- SD. Individual thresholds for each odorant are denoted with open triangles. Upward triangles denote males while downward triangles denote females. Letters signify statistical differences between two thresholds (p<0.05, two-way ANOVA with multiple comparisons). Statistical significance for these pairwise comparisons is displayed in both directions.

https://doi.org/10.1371/journal.pone.0298448.g003

way ANOVA with multiple comparisons and Bonferroni correction). A power analysis determined that a group size of 32–48 animals (16–24 males and 16–24 females) per odor would be necessary to potentially observe statistical significance in odor sensitivity between sexes for specific odorants.

Of all the odorants tested, mice exhibited the greatest sensitivity to the acyclic monoterpene, geraniol. The average vapor-phase detection threshold to this odorant was 0.70 ppb (95% CI: 0.49–1.05 ppb), which is equivalent to a 1.02 x 10^{-5} v/v (95% CI: 0.62–1.78 x 10^{-5} v/v) dilution of geraniol in mineral oil (Figs 2A and 3). Individual mice differed in their sensitivity to this odorant by less than one order of magnitude (0.46–2.22 ppb). Mice were more sensitive to geraniol than other tested acyclic monoterpenes (citral $p < 0.001$; linalool $p < 0.001$), monocylic monoterpenes (γ-terpinene $p < 0.001$; r-limonene $p = 0.008$; s-limonene $p = 0.002$), and a single bicyclic monoterpene (β-pinene $p < 0.001$) (DF = 49; two-way ANOVA with multiple comparisons and Bonferroni correction) (Fig 3).

The average vapor-phase detection threshold for citral was 16.08 ppb (95% CI: 7.60–42.51 ppb), which is equivalent to a 4.51 x 10^{-5} v/v (95% CI: 2.18–11.71 x 10^{-5} v/v) dilution in mineral oil (Figs 2B and 3). Individual mice differed in their sensitivity to this odorant by more than one order of magnitude (4.39–83.37 ppb). Mice were less sensitive to citral than geraniol ($p < 0.001$) and two bicyclic monoterpenes (α-pinene $p = 0.014$ and eucalyptol

p = 0.002) (DF = 49; two-way ANOVA with multiple comparisons and Bonferroni correction) (Fig 3).

The average vapor-phase detection threshold for linalool was 16.94 ppb (95% CI: 8.09–44.10 ppb), which is equivalent to a 1.74 x 10^{-5} v/v (95% CI: 0.77–4.98 x 10^{-5} v/v) liquid dilution in mineral oil (Figs 2C and 3). Individual mice differed in their sensitivity to this odorant by more than one order of magnitude (3.94–59.66 ppb). Mice were less sensitive to linalool than geraniol ($p<0.001$), two bicyclic monoterpenes (α-pinene $p = 0.005$ and eucalyptol $p = 0.001$) (DF = 49; two-way ANOVA with multiple comparisons and Bonferroni correction) (Fig 3).

Of the monocyclic monoterpenes tested, mice were most sensitive to s-limonene. The average vapor-phase detection threshold to this odorant was 5.01 ppb (95% CI: 2.78–9.13 ppb), which is equivalent to a 5.40 x 10^{-6} v/v (95% CI: 2.63–10.99 x 10^{-6} v/v) dilution of s-limonene in mineral oil (Figs 2D and 3). Individual mice differed in their sensitivity to this odorant by more than one order of magnitude (0.60–38.5 ppb). Mice were only less sensitive to s-limonene as compared to geraniol ($p = 0.002$; DF = 49; two-way ANOVA with multiple comparisons and Bonferroni correction) (Fig 3).

Mice displayed a similar sensitivity to its enantiomer, r-limonene. The average vapor-phase detection threshold to this odorant was 5.44 ppb (95% CI: 2.89–11.63 ppb), which is equivalent to a 9.17 x 10^{-6} v/v (95% CI: 4.68–20.58 x 10^{-6} v/v) dilution of r-limonene in mineral oil (Figs 2E and 3). Individual mice differed in their sensitivity to this odorant by more than one order of magnitude (0.69–17.79 ppb). Mice were only less sensitive to r-limonene as compared to geraniol ($p = 0.008$; DF = 49; two-way ANOVA with multiple comparisons and Bonferroni correction) (Fig 3).

Among the monocyclic monoterpenes tested, mice were least sensitive to γ-terpinene. The average vapor-phase detection threshold to this odorant was 18.10 ppb (95% CI: 9.18–36.73 ppb), which is equivalent to a 2.11 x 10^{-5} v/v (95% CI: 0.97–4.71 x 10^{-5} v/v) dilution of γ-terpinene in mineral oil (Figs 2F and 3). Individual mice differed in their sensitivity to this odorant by more than one order of magnitude (3.30–63.32 ppb). Mice were less sensitive to γ-terpinene as compared to the geraniol ($p < 0.0001$) and eucalyptol (p = 0.024) (DF = 49; two-way ANOVA with multiple comparisons and Bonferroni correction) (Fig 3).

Among the bicyclic monoterpenes tested, mice were most sensitive to eucalyptol. The average vapor-phase detection threshold to this odorant was 1.97 ppb (95% CI: 1.20–3.34 ppb), which is equivalent to a 3.97 x 10^{-6} v/v (95% CI: 2.40–6.75 x 10^{-6} v/v) dilution of eucalyptol in mineral oil (Figs 2G and 3). Individual mice differed in their sensitivity to this odorant by less than one order of magnitude (0.52–5.07 ppb). Mice were more sensitive to eucalyptol than β-pinene ($p = 0.0003$), γ-terpinene ($p = 0.014$), and citral ($p = 0.002$) (DF = 49; two-way ANOVA with multiple comparisons and Bonferroni correction) (Fig 3).

The average vapor-phase detection threshold to α-pinene was 2.45 ppb (95% CI: 1.49–4.05 ppb), which is equivalent to an 8.87 x 10^{-7} v/v (95% CI: 5.01–15.51 x 10^{-7} v/v) dilution in mineral oil (Figs 2H and 3). Individual mice differed in their sensitivity to this odorant by more than one order of magnitude (0.35–11.78 ppb). Mice were more sensitive to α-pinene than its isomer β-pinene ($p = 0.002$), and the acyclic monoterpenes citral (p = 0.014) and linalool (p = 0.005) (DF = 49; two-way ANOVA with multiple comparisons and Bonferroni correction) (Fig 3).

Among the bicyclic monoterpenes tested, mice were least sensitive to β-pinene. The average vapor-phase detection threshold to this odorant was 17.80 ppb (95% CI: 12.95–25.82 ppb), which is equivalent to a 1.23 x 10^{-5} v/v (95% CI: 0.87–1.86 x 10^{-5} v/v) dilution of β-pinene in mineral oil (Figs 2I and 3). Individual mice differed in their sensitivity to this odorant by less than one order of magnitude (12.18–48.62 ppb). Mice were less sensitive to β-pinene than its

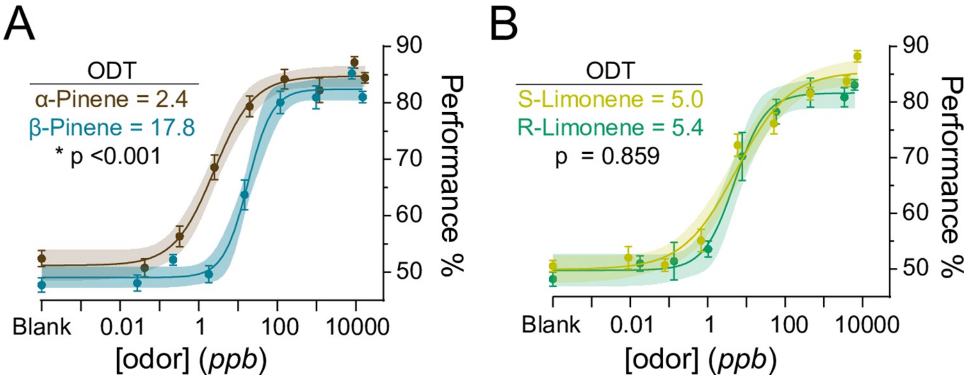

Fig 4. C57BL/6J mice are differentially sensitive to pinene isomers but not limonene enantiomers. Psychometric curves comparing α-pinene and β-pinene (**A**), as well as r-limonene and s-limonene (**B**). Data were fitted using a hill function. Maximal behavioral performance for each odor concentration is limited to ~85% (see methods). Plots show mean +/- SE with shaded 95% confidence interval. Statistical significance was calculated using a sum of squares F-test.

https://doi.org/10.1371/journal.pone.0298448.g004

isomer α-pinene (p = 0.002), as well as eucalyptol (p = 0.0003) and geraniol ($p < 0.0001$) (DF = 49; two-way ANOVA with multiple comparisons and Bonferroni correction) (Fig 3).

We also compared the average behavioral detection thresholds for the bicyclic monoterpene isomers: α-pinene and β-pinene, and the monocyclic monoterpene enantiomers: r-limonene and s-limonene. As stated above, mice were statistically more sensitive to α-pinene than β-pinene ($p < 0.001$, F = 38.55, sum of squares F-test; Fig 4A). In contrast, mice display similar sensitivities to r-limonene and s-limonene (p = 0.8599, F = 0.0313, sum of squares F-test; Fig 4B).

We did not find a statistically significant correlation between our monoterpene detection thresholds and either the atmospheric lifetime (r = 0.0418; p = 0.9221, Spearman correlation), volatility (r = -0.2594; p = 0.4980, Spearman correlation), or the air/mucus partition coefficient (r = 0.0084; p = 0.9915, Spearman correlation) of the odorants.

To estimate the maximal concentrations of monoterpenes naturally experienced by mice, we pooled data from studies analyzing the headspace concentration of monoterpenes emitted by various sources, including fruits, vegetables, nuts, and plants. For the majority of sources tested, monoterpene source concentrations ranged from ~1–1000 ppb, with a maximum of ~80 ppm (Fig 5A). In most cases, these source concentrations exceeded our measures of mouse sensitivity, indicating that mice can detect most (but not all) of the monoterpene volatiles emitted from these sources.

Lastly, to provide further context to our estimations of monoterpene sensitivity, we have plotted all the human thresholds for these odorants compiled by Gemert, 2011 [37] (Fig 5B). Our estimations of murine sensitivity for β-pinene and γ-terpinene are lower than all the compiled human thresholds. For other monoterpenes, including geraniol, limonene, eucalyptol, and α-pinene, our estimations of threshold in mice are lower than most, but not all, of the compiled human thresholds. In contrast, humans appear to be more sensitive to citral and potentially linalool. Despite the large discrepancies in estimations of human thresholds across studies, we find that mice have roughly similar sensitivities to these odorants as humans.

Discussion

We found that C57BL/6J mice can reliably detect monoterpene concentrations in the parts per billion range. On average, mice are most sensitive to geraniol and least sensitive to γ-terpinene. We did not observe any statistically significant relationship between monoterpene sensitivity

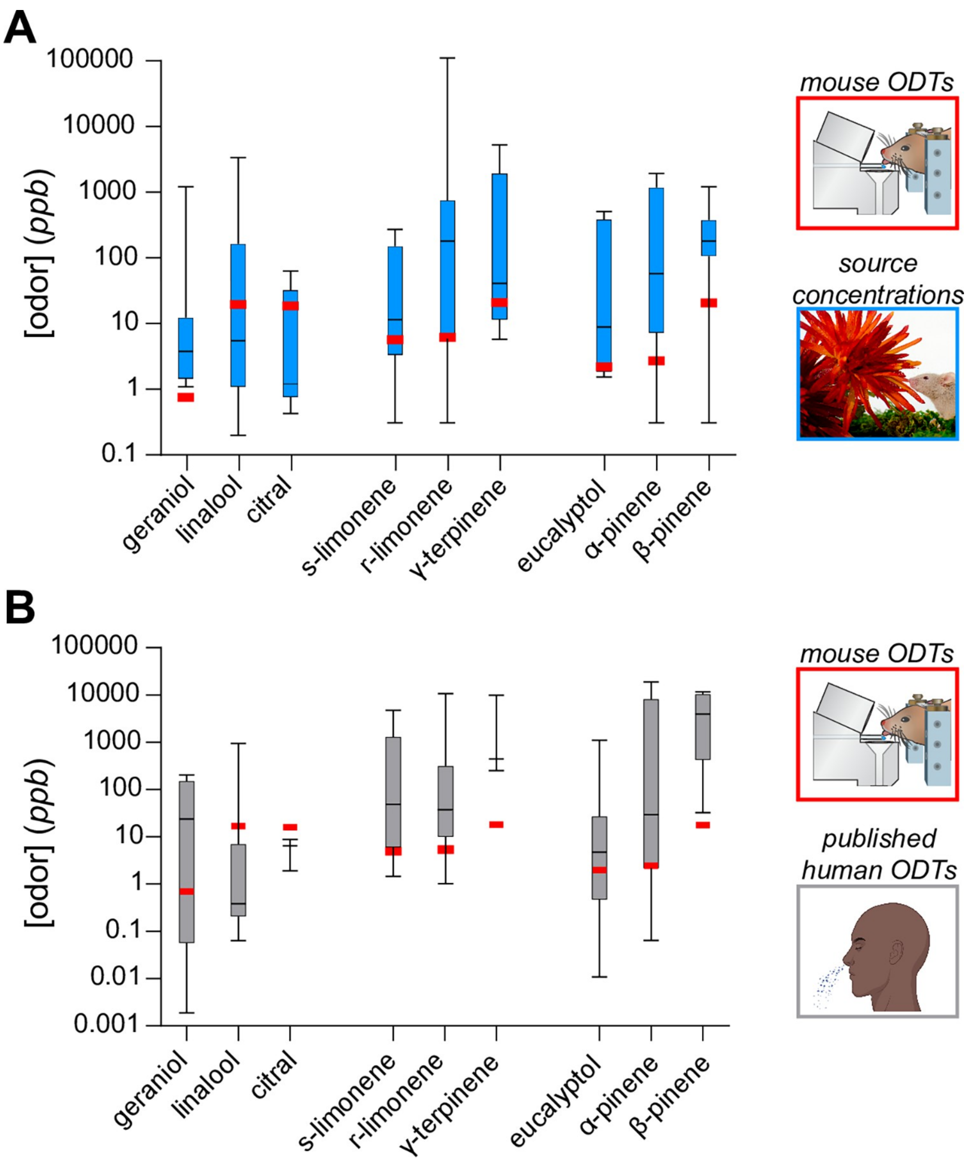

Fig 5. Contextualizing our estimations of monoterpene sensitivity in mice using published source concentrations and human detection thresholds. (**A**) Box and whisker plot of monoterpene concentrations emitted by sources relevant to mice, including various fruits, vegetables, nuts, and plants (see methods). All concentrations were determined using headspace volatile analysis via gas chromatography/mass spectrometry (GC/MS) or proton transfer reaction mass spectrometry (PTR-MS). (**B**) Box and whisker plot of human detection thresholds to monoterpenes compiled by Gemert, 2011. Red bars signify our estimation of the murine detection threshold for each odorant. Published human ODT icon was created with Biorender.com.

https://doi.org/10.1371/journal.pone.0298448.g005

and either volatility, air/mucous coefficient, or the atmospheric lifetime of an odorant. We found that mice were more sensitive to α-pinene than its isomer, β-pinene, but exhibited similar thresholds to the limonene enantiomers. These robust estimations of monoterpene sensitivity define the minimum concentrations that should be utilized in functional studies and provide context for the vapor-phase delivery of these chemicals in studies investigating their biological activity in mice.

Our estimates of monoterpene sensitivity represent the first behavioral threshold measurements for six out of the nine odorants in mice. A previous study examining the contribution of

NKCC1 found that wild-type mice exhibited a eucalyptol threshold of 242 ppb [19] (once we corrected the data for solvent effects using published equilibrium equations [35]). In contrast, we found that mice were approximately 100-fold more sensitive to this odorant, exhibiting a threshold of 2 ppb. As we have noted previously [22, 23], measurements of olfactory sensitivity can be influenced by a number of experimental factors including mouse strain, the definition of threshold, whether the animal is head-fixed or freely-moving, the method of odor delivery, and the training/testing procedure [60–63]. Please see Williams and Dewan (2020) [22], for a detailed discussion of our approach and how it compares to previous methods.

The sensitivity of mice to r-limonene and s-limonene has also been examined, but published thresholds dramatically differ. Blount and Coppola (2020) reported a solvent-corrected r-limonene threshold of 6.2 x 10^{-14} ppb (or 6.2 parts per septillion) in CD-1 mice [18]. In contrast, Joshi et al., (2006) tested the same strain of mice and found an average r-limonene threshold that is approximately 15 orders of magnitude higher (~117 ppb, once we corrected the data for solvent effects) [17]. Our estimation of the r-limonene sensitivity in C57BL/6J mice was more similar to the second study, yielding a threshold of 5.4 ppb. Further, Joshi et al., (2006) reported an average s-limonene threshold of 2.1 ppb (once corrected for solvent effects) [17], which was similar to our sensitivity measurement in C57BL/6J mice of 5.0 ppb. It should be noted that our mice are also highly trained and experience a very high degree of task replication, similar to the Blount and Coppola study [18], but exhibit more moderate thresholds. Thus, we would recommend that the minimum monoterpene concentrations utilized for functional studies, in mice, should be in the ppb range.

Monoterpene enantiomers are commonly used to probe the behavioral limits of odor discrimination [17, 18, 64–68], but have only been rarely examined in the context of sensitivity. As mentioned above, a previous study found CD-1 mice were more sensitive to s-limonene than r-limonene [17]. Although our estimation of s-limonene sensitivity in C57Bl/6J mice was similar to this previous study (2.1 vs 5.0 ppb), we did not observe any statistical difference in sensitivity to its enantiomer, r-limonene (5.4 ppb). It is possible that this discrepancy relates to strain differences in the receptor repertoire, but further research is needed. It is interesting to note that humans also exhibit similar thresholds for these enantiomers [69]. However, humans, rats, and mice [17, 18, 64, 70] can easily discriminate between these odorants, highlighting that despite similar sensitivities, their respective neural response is unique at least for the concentrations tested.

In addition to the main olfactory system, the trigeminal system can detect airborne chemicals and thus has the potential to impact sensitivity [71]. While the contribution of the trigeminal system to monoterpene detection has not been investigated in mice, a study utilizing anosmic humans found that geraniol, α-pinene, β-pinene, and both limonene enantiomers did not elicit any nasal pungency, while trigeminal sensitivity to citral and linalool was greater than three orders of magnitude higher than olfactory sensitivity [69]. Thus, it seems unlikely that mice are using their trigeminal system to enhance their sensitivity to monoterpenes, but further research is needed.

Our olfactory threshold measurements and published source concentrations can provide a guideline for experimenters to choose appropriate stimulus concentrations for functional studies. Based on our results, we would suggest a lower limit of ~20 ppb, as this concentration is above the detection threshold for all tested monoterpenes. It is important to note that one cannot infer this lower limit based solely on source concentrations, as the levels of specific odorants emitted by a natural source can be below an animal's detection threshold. In fact, Dunkel et al., 2014 concluded that as few as 3% of the volatile chemicals emitted from a food source contribute to its specific smell [72]. Published source concentrations can be used to estimate the maximal stimulus concentration that a mouse could encounter in nature and thus,

theoretically set the upper limit for relevant stimulus intensities. However, one clear caveat to our dataset is the limited number of studies that examine odorous sources within the natural environment of a mouse. Despite this shortcoming, we suggest an upper limit of ~1000 ppb for these monoterpenes. In conclusion, we would suggest utilizing vapor-phase monoterpene concentrations within the range of 20 to 1000 ppb for functional studies in mice.

Perceptual measures of sensitivity provide a mechanism to compare the olfactory system of different species. In fact, there is significant interest in how the olfactory capabilities of humans compares to a macrosmatic species like mice [73–77]. We found that the range of published human thresholds, albeit from a single collated source [37], were relatively similar to our sensitivity measures in mice. However, it is important to note that our estimations of murine sensitivity frequently fell below many of the median published human thresholds and mice appear to be more sensitive to both γ-terpinene and β-pinene. Despite these differences, the overall similarity between human and mouse sensitivity may indicate that experimenters may be able to use their own olfactory system to assess the quality of their odor delivery system, including testing for monoterpene contamination.

Using the same method, we have previously measured the sensitivity of mice to aliphatic alcohols [22], esters [20, 21, 23], and amines [21]. We find that in general, mice are more sensitive to alcohols than these other odorants (Fig 6). Alcohols, carbonyls, and hydrocarbons have been identified as key volatiles produced by grain spoilage fungi [78]. The enhanced sensitivity of mice to alcohols could potentially assist in the identification of spoiled grains, which is a major food source for both wild and laboratory mice [79]. Likewise, the enhanced sensitivity

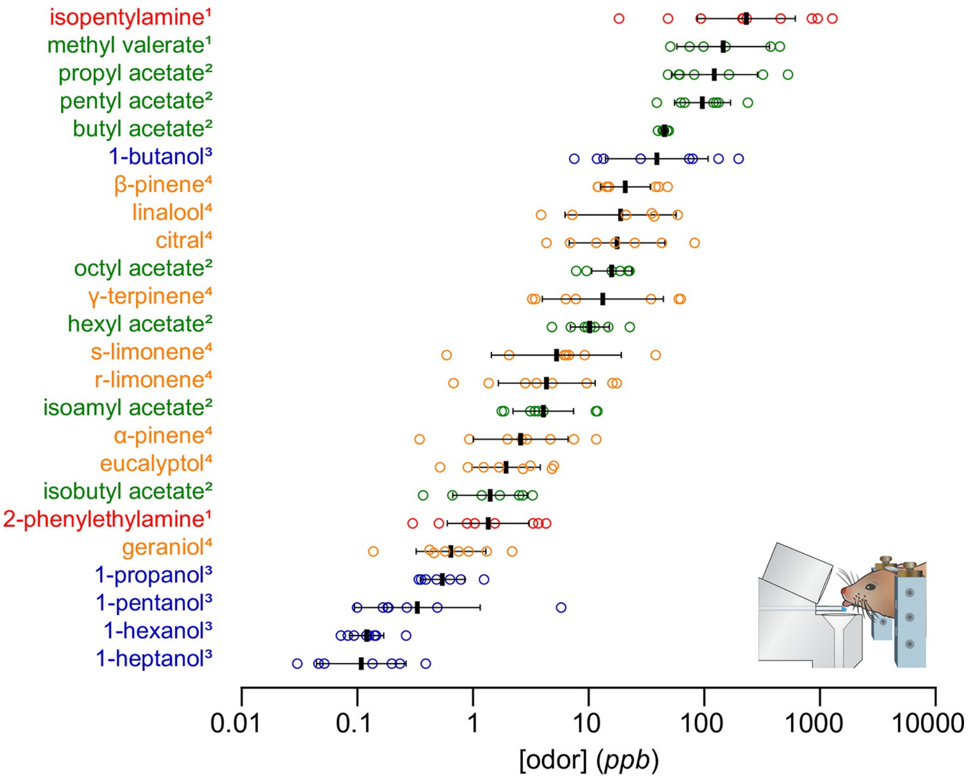

Fig 6. Summary of olfactory detection thresholds measured with the same approach. Plots show mean +/- SD. Individual thresholds for each odorant are denoted with open circles with the color denoting the chemical class (red–amine; green–ester, orange–monoterpene, blue–alcohol). Detection thresholds were corrected for solvent effects and obtained from 1—Dewan et al., (2018); 2- Jennings et al., (2022); 3- Williams and Dewan (2020); 4-current study.

https://doi.org/10.1371/journal.pone.0298448.g006

to 2-phenylethylamine likely relates to its putative role as a predator odor [80, 81]. The high sensitivity to various monoterpenes, including geraniol, eucalyptol, and α-pinene was surprising, as these odorants have no known social relevance. Monoterpenes are among several classes of chemicals that are produced by plants for the purpose of chemical communication including attracting pollinators [82] and signaling damage from herbivores [83]. It is therefore possible that mice are eavesdropping on these volatile cues to detect potential food sources for their omnivorous diet, such herbivorous arthropods [84]. Interestingly, monoterpenes have also been found to have medicinal effects in both humans and mice [2, 5–12]; however, to our knowledge, there is no evidence of mice actively seeking natural sources of these chemicals.

Conclusions

We have provided robust measurements of sensitivity in C57BL/6J mice to a series of acyclic, monocyclic, and bicyclic monoterpenes. These estimates serve to set the lower limit of monoterpene concentrations that should be used in functional studies. In addition, we have attempted to define an upper limit for these stimulus intensities by collating published measurements of monoterpene concentrations emitted from natural sources. Based on these two complementary datasets, we would suggest utilizing vapor-phase concentrations of these monoterpenes within the range of 20 to 1000 ppb for functional studies in mice. It is our hope that these analyses will help researchers use appropriate concentrations for functional studies employing monoterpenes and provide context for the vapor-phase delivery of these odorants to investigate their biological activity in rodent models.

Supporting information

S1 Fig. Photoionization device (PID) traces of 250 sequential stimulus presentations from a single vial of the acyclic monoterpenes: geraniol (**A**), citral (**B**), and linalool (**C**), the monocyclic monoterpenes: s-limonene (**D**), r-limonene (**E**), and γ-terpinene (**F**), and the bicyclic monoterpenes: eucalyptol (**G**), α-pinene (**H**), and β-pinene (**I**). Shaded area signifies 2 second stimulus period.
(TIF)

Acknowledgments

We thank Chloe Johnson, Sam Caton, Fernanda Antezana, and Aparna Prasad for their support on this project; Fred Fletcher and Te Tang for their help building the operant conditioning rigs as well as coding the Python software; Enrique Cometto-Muniz for advice with odorant / solvent interactions, and Charles Badland for graphic assistance.

Author Contributions

Conceptualization: Ellie Williams, Adam Dewan.

Data curation: Ellie Williams, Austin Pauley, Adam Dewan.

Formal analysis: Ellie Williams, Austin Pauley, Adam Dewan.

Funding acquisition: Adam Dewan.

Investigation: Ellie Williams.

Project administration: Adam Dewan.

Supervision: Adam Dewan.

Writing – original draft: Ellie Williams, Austin Pauley, Adam Dewan.

Writing – review & editing: Ellie Williams, Austin Pauley, Adam Dewan.

References

1. Rosenkranz M, Chen Y, Zhu P, Vlot AC. Volatile terpenes–mediators of plant-to-plant communication. Plant J. 2021; 108: 617–631. https://doi.org/10.1111/tpj.15453 PMID: 34369010
2. Wojtunik-Kulesza KA, Kasprzak K, Oniszczuk T, Oniszczuk A. Natural Monoterpenes: Much More than Only a Scent. Chem Biodivers. 2019; 16: e1900434. https://doi.org/10.1002/cbdv.201900434 PMID: 31587473
3. Zielińska-Błajet M, Feder-Kubis J. Monoterpenes and Their Derivatives—Recent Development in Biological and Medical Applications. Int J Mol Sci. 2020; 21: 7078. https://doi.org/10.3390/ijms21197078 PMID: 32992914
4. De Alvarenga JFR, Genaro B, Costa BL, Purgatto E, Manach C, Fiamoncini J. Monoterpenes: current knowledge on food source, metabolism, and health effects. Crit Rev Food Sci Nutr. 2023; 63: 1352–1389. https://doi.org/10.1080/10408398.2021.1963945 PMID: 34387521
5. Salakhutdinov NF, Volcho KP, Yarovaya OI. Monoterpenes as a renewable source of biologically active compounds. Pure Appl Chem. 2017; 89: 1105–1117. https://doi.org/10.1515/pac-2017-0109
6. Gomes PB, Feitosa ML, Silva MIG, Noronha EC, Moura BA, Venâncio ET, et al. Anxiolytic-like effect of the monoterpene 1,4-cineole in mice. Pharmacol Biochem Behav. 2010; 96: 287–293. https://doi.org/10.1016/j.pbb.2010.05.019 PMID: 20670917
7. Satou T, Kasuya H, Maeda K, Koike K. Daily Inhalation of α-Pinene in Mice: Effects on Behavior and Organ Accumulation. Phytother Res. 2014; 28: 1284–1287. https://doi.org/10.1002/ptr.5105 PMID: 25340185
8. Harada H, Kashiwadani H, Kanmura Y, Kuwaki T. Linalool Odor-Induced Anxiolytic Effects in Mice. Front Behav Neurosci. 2018; 12: 241. https://doi.org/10.3389/fnbeh.2018.00241 PMID: 30405369
9. Lima NGPB, De Sousa DP, Pimenta FCF, Alves MF, De Souza FS, Macedo RO, et al. Anxiolytic-like activity and GC–MS analysis of (R)-(+)-limonene fragrance, a natural compound found in foods and plants. Pharmacol Biochem Behav. 2013; 103: 450–454. https://doi.org/10.1016/j.pbb.2012.09.005 PMID: 22995322
10. Sousa DPD, Raphael E, Brocksom U, Brocksom TJ. Sedative Effect of Monoterpene Alcohols in Mice: A Preliminary Screening. Z Für Naturforschung C. 2007; 62: 563–566. https://doi.org/10.1515/znc-2007-7-816 PMID: 17913072
11. Do Amaral JF, Silva MIG, De Aquino Neto MRA, Neto PFT, Moura BA, De Melo CTV, et al. Antinociceptive Effect of the Monoterpene R-(+)-Limonene in Mice. Biol Pharm Bull. 2007; 30: 1217–1220. https://doi.org/10.1248/bpb.30.1217 PMID: 17603156
12. Amaral MPDMD, Silva Junior MPD, Lima FDCA, Gutierrez SJCArcanjo DDR, Oliveira RDCM. Anxiolytic/Sedative Effect of Monoterpene (–)-Borneol in Mice and In Silico Molecular Interaction with GABAA Receptor. Future Pharmacol. 2023; 3: 132–141. https://doi.org/10.3390/futurepharmacol3010009
13. Reis S, Mantello A, Macedo J, Gelfuso E, Da Silva C, Fachin A, et al. Typical Monoterpenes as Insecticides and Repellents against Stored Grain Pests. Molecules. 2016; 21: 258. https://doi.org/10.3390/molecules21030258 PMID: 26907246
14. Abdelgaleil SAM, Gad HA, Ramadan GRM, El-Bakry AM, El-Sabrout AM. Monoterpenes: chemistry, insecticidal activity against stored product insects and modes of action—a review. Int J Pest Manag. 2021; 1–23. https://doi.org/10.1080/09670874.2021.1982067
15. Habtemariam S. Antidiabetic Potential of Monoterpenes: A Case of Small Molecules Punching above Their Weight. Int J Mol Sci. 2017; 19: 4. https://doi.org/10.3390/ijms19010004 PMID: 29267214
16. Wüst M. Biosynthesis of Plant-Derived Odorants. In: Buettner A, editor. Springer Handbook of Odor. Cham: Springer International Publishing; 2017. pp. 9–10. https://doi.org/10.1007/978-3-319-26932-0_2
17. Joshi D, Völkl M, Shepherd GM, Laska M. Olfactory sensitivity for enantiomers and their racemic mixtures—a comparative study in CD-1 mice and spider monkeys. Chem Senses. 2006; 31: 655–664. https://doi.org/10.1093/chemse/bjl006 PMID: 16793858
18. Blount A, Coppola DM. The effect of odor enrichment on olfactory acuity: Olfactometric testing in mice using two mirror-molecular pairs. PloS One. 2020; 15: e0233250. https://doi.org/10.1371/journal.pone.0233250 PMID: 32730274

19. Smith DW, Thach S, Marshall EL, Mendoza M-G, Kleene SJ. Mice lacking NKCC1 have normal olfactory sensitivity. Physiol Behav. 2008; 93: 44–49. https://doi.org/10.1016/j.physbeh.2007.07.011 PMID: 17719611

20. Johnson CE, Hammock EAD, Dewan AK. Vasopressin receptor 1a, oxytocin receptor, and oxytocin knockout male and female mice display normal perceptual abilities towards non-social odorants. Horm Behav. 2023; 148: 105302. https://doi.org/10.1016/j.yhbeh.2022.105302 PMID: 36628861

21. Dewan A, Cichy A, Zhang J, Miguel K, Feinstein P, Rinberg D, et al. Single olfactory receptors set odor detection thresholds. Nat Commun. 2018; 9: 2887. https://doi.org/10.1038/s41467-018-05129-0 PMID: 30038239

22. Williams E, Dewan A. Olfactory Detection Thresholds for Primary Aliphatic Alcohols in Mice. Chem Senses. 2020. https://doi.org/10.1093/chemse/bjaa045 PMID: 32609815

23. Jennings L, Williams E, Avlas M, Dewan A. The behavioral sensitivity of mice to acetate esters. Chem Senses. 2022; 47: bjac017. https://doi.org/10.1093/chemse/bjac017 PMID: 35816188

24. Johnson CE, Williams E, Dewan A. Protocol for quantifying the odor detection threshold of mice. STAR Protoc. 2023; 4: 102635. https://doi.org/10.1016/j.xpro.2023.102635 PMID: 37805920

25. Burton SD, Brown A, Eiting TP, Youngstrom IA, Rust TC, Schmuker M, et al. Mapping odorant sensitivities reveals a sparse but structured representation of olfactory chemical space by sensory input to the mouse olfactory bulb. eLife. 2022; 11: e80470. https://doi.org/10.7554/eLife.80470 PMID: 35861321

26. Linster C, Johnson BA, Yue E, Morse A, Xu Z, Hingco EE, et al. Perceptual Correlates of Neural Representations Evoked by Odorant Enantiomers. J Neurosci. 2001; 21: 9837–9843. https://doi.org/10.1523/JNEUROSCI.21-24-09837.2001 PMID: 11739591

27. Li Y, Swerdloff M, She T, Rahman A, Sharma N, Shah R, et al. Robust odor identification in novel olfactory environments in mice. Nat Commun. 2023; 14: 673. https://doi.org/10.1038/s41467-023-36346-x PMID: 36781878

28. Radvansky BA, Dombeck DA. An olfactory virtual reality system for mice. Nat Commun. 2018; 9: 839. https://doi.org/10.1038/s41467-018-03262-4 PMID: 29483530

29. Poo C, Isaacson JS. Odor representations in olfactory cortex: "sparse" coding, global inhibition, and oscillations. Neuron. 2009; 62: 850–861. https://doi.org/10.1016/j.neuron.2009.05.022 PMID: 19555653

30. Pashkovski SL, Iurilli G, Brann D, Chicharro D, Drummey K, Franks KM, et al. Structure and flexibility in cortical representations of odour space. Nature. 2020; 583: 253–258. https://doi.org/10.1038/s41586-020-2451-1 PMID: 32612230

31. Gadziola MA, Tylicki KA, Christian DL, Wesson DW. The olfactory tubercle encodes odor valence in behaving mice. J Neurosci Off J Soc Neurosci. 2015; 35: 4515–4527. https://doi.org/10.1523/JNEUROSCI.4750-14.2015 PMID: 25788670

32. Parabucki A, Bizer A, Morris G, Munoz AE, Bala ADS, Smear M, et al. Odor Concentration Change Coding in the Olfactory Bulb. eneuro. 2019; 6: ENEURO.0396–18.2019. https://doi.org/10.1523/ENEURO.0396-18.2019 PMID: 30834303

33. Scott JW, Sherrill L, Jiang J, Zhao K. Tuning to odor solubility and sorption pattern in olfactory epithelial responses. J Neurosci Off J Soc Neurosci. 2014; 34: 2025–2036. https://doi.org/10.1523/JNEUROSCI.3736-13.2014 PMID: 24501345

34. Williams J, Ringsdorf A. Human odour thresholds are tuned to atmospheric chemical lifetimes. Philos Trans R Soc B Biol Sci. 2020; 375: 20190274. https://doi.org/10.1098/rstb.2019.0274 PMID: 32306881

35. Cometto-Muñiz JE, Cain WS, Abraham MH. Quantification of chemical vapors in chemosensory research. Chem Senses. 2003; 28: 467–477. https://doi.org/10.1093/chemse/28.6.467 PMID: 12907584

36. Jennings L, Williams E, Caton S, Avlas M, Dewan A. Estimating the relationship between liquid and vapor-phase odorant concentrations using a photoionization detector (PID) based approach. Chem Senses. 2022; bjac038. https://doi.org/10.1093/chemse/bjac038 PMID: 36571813

37. Gemert LJ van. Odour thresholds: Compilations of odour thresholds values in air, water and other media (Edition 2011). Utrecht: Oliemans Punter & Partners BV; 2011.

38. Alasalvar C. Method for the static headspace analysis of carrot volatiles. Food Chem. 1999; 65: 391–397. https://doi.org/10.1016/S0308-8146(98)00202-7

39. Farneti B, Cristescu SM, Costa G, Harren FJM, Woltering EJ. Rapid Tomato Volatile Profiling by Using Proton-Transfer Reaction Mass Spectrometry (PTR-MS). J Food Sci. 2012; 77. https://doi.org/10.1111/j.1750-3841.2012.02679.x PMID: 22509736

40. Iqbal M, Kim K-H, Ahn J. Monoterpenes Released from Fruit, Plant, and Vegetable Systems. Sensors. 2014; 14: 18286–18301. https://doi.org/10.3390/s141018286 PMID: 25268921

41. Zhou A, McFeeters RF. Volatile Compounds in Cucumbers Fermented in Low-Salt Conditions. J Agric Food Chem. 1998; 46: 2117–2122. https://doi.org/10.1021/jf9704726

42. Farneti B, Khomenko I, Grisenti M, Ajelli M, Betta E, Algarra AA, et al. Exploring Blueberry Aroma Complexity by Chromatographic and Direct-Injection Spectrometric Techniques. Front Plant Sci. 2017; 8: 617. https://doi.org/10.3389/fpls.2017.00617 PMID: 28491071

43. Bianchi T, Weesepoel Y, Koot A, Iglesias I, Eduardo I, Gratacós-Cubarsí M, et al. Investigation of the aroma of commercial peach (Prunus persica L. Batsch) types by Proton Transfer Reaction–Mass Spectrometry (PTR-MS) and sensory analysis. Food Res Int. 2017; 99: 133–146. https://doi.org/10.1016/j.foodres.2017.05.007 PMID: 28784469

44. Ceccarelli A, Farneti B, Khomenko I, Cellini A, Donati I, Aprea E, et al. Nectarine volatilome response to fresh-cutting and storage. Postharvest Biol Technol. 2020; 159: 111020. https://doi.org/10.1016/j.postharvbio.2019.111020

45. Farneti B, Khomenko I, Cappellin L, Ting V, Romano A, Biasioli F, et al. Comprehensive VOC profiling of an apple germplasm collection by PTR-ToF-MS. Metabolomics. 2015; 11: 838–850. https://doi.org/10.1007/s11306-014-0744-9

46. Taiti C, Giordani E, Palm E, Petrucci AW, Bennati G, Gestri G, et al. Volatile compounds from different fruit parts of two cultivars of Cydonia oblonga. Adv Hortic Sci. 2018; 105–111 Pages. https://doi.org/10.13128/AHS-22807

47. Wu Y, Pan Q, Qu W, Duan C. Comparison of Volatile Profiles of Nine Litchi (*Litchi chinensis* Sonn.) Cultivars from Southern China. J Agric Food Chem. 2009; 57: 9676–9681. https://doi.org/10.1021/jf902144c PMID: 19803519

48. Sun H, Chen W, Jiang Y, He Q, Li X, Guo Q, et al. Characterization of volatiles in red- and white-fleshed loquat (Eriobotrya japonica) fruits by electronic nose and headspace solid-phase microextraction with gas chromatography-mass spectrometry. Food Sci Technol. 2020; 40: 21–32. https://doi.org/10.1590/fst.27318

49. Kim Y-H, Kim K-H, Szulejko J, Parker D. Quantitative Analysis of Fragrance and Odorants Released from Fresh and Decaying Strawberries. Sensors. 2013; 13: 7939–7978. https://doi.org/10.3390/s130607939 PMID: 23787728

50. Różańska A, Sieńska D, Dymerski T, Namieśnik J. Analysis of volatile fraction of sweetie (Citrus maxima × Citrus paradisi) and its parent fruit using proton transfer reaction mass spectrometry. Monatshefte Chem. 2018; 149: 1629–1634. https://doi.org/10.1007/s00706-018-2229-4 PMID: 30174350

51. Ghanbari J, Khajoei-Nejad G, Erasmus SW, Van Ruth SM. Identification and characterisation of volatile fingerprints of saffron stigmas and petals using PTR-TOF-MS: Influence of nutritional treatments and corm provenance. Ind Crops Prod. 2019; 141: 111803. https://doi.org/10.1016/j.indcrop.2019.111803

52. Aprea E, Biasioli F, Carlin S, Versini G, Märk TD, Gasperi F. Rapid white truffle headspace analysis by proton transfer reaction mass spectrometry and comparison with solid-phase microextraction coupled with gas chromatography/mass spectrometry. Rapid Commun Mass Spectrom RCM. 2007; 21: 2564–2572. https://doi.org/10.1002/rcm.3118 PMID: 17639568

53. Di Guardo M, Farneti B, Khomenko I, Modica G, Mosca A, Distefano G, et al. Genetic characterization of an almond germplasm collection and volatilome profiling of raw and roasted kernels. Hortic Res. 2021; 8: 27. https://doi.org/10.1038/s41438-021-00465-7 PMID: 33518710

54. Yener S, Sánchez-López JA, Granitto PM, Cappellin L, Märk TD, Zimmermann R, et al. Rapid and direct volatile compound profiling of black and green teas (Camellia sinensis) from different countries with PTR-ToF-MS. Talanta. 2016; 152: 45–53. https://doi.org/10.1016/j.talanta.2016.01.050 PMID: 26992494

55. Silvis ICJ, Luning PA, Klose N, Jansen M, Van Ruth SM. Similarities and differences of the volatile profiles of six spices explored by Proton Transfer Reaction Mass Spectrometry. Food Chem. 2019; 271: 318–327. https://doi.org/10.1016/j.foodchem.2018.07.021 PMID: 30236683

56. Acierno V, De Jonge L, Van Ruth S. Sniffing out cocoa bean traits that persist in chocolates by PTR-MS, ICP-MS and IR-MS. Food Res Int. 2020; 133: 109212. https://doi.org/10.1016/j.foodres.2020.109212 PMID: 32466947

57. Portillo-Estrada M, Van Moorleghem C, Janssenswillen S, Cooper RJ, Birkemeyer C, Roelants K, et al. Proton-transfer-reaction time-of-flight mass spectrometry (PTR-TOF-MS) as a tool for studying animal volatile organic compound (VOC) emissions. Durand P, editor. Methods Ecol Evol. 2021; 12: 748–766. https://doi.org/10.1111/2041-210X.13554

58. Yan J, Alewijn M, Van Ruth SM. From Extra Virgin Olive Oil to Refined Products: Intensity and Balance Shifts of the Volatile Compounds versus Odor. Molecules. 2020; 25: 2469. https://doi.org/10.3390/molecules25112469 PMID: 32466443

59. Pico J, Khomenko I, Capozzi V, Navarini L, Biasioli F. Real-Time Monitoring of Volatile Compounds Losses in the Oven during Baking and Toasting of Gluten-Free Bread Doughs: A PTR-MS Evidence. Foods. 2020; 9: 1498. https://doi.org/10.3390/foods9101498 PMID: 33092071

60. Bodyak N, Slotnick B. Performance of mice in an automated olfactometer: odor detection, discrimination and odor memory. Chem Senses. 1999; 24: 637–645. https://doi.org/10.1093/chemse/24.6.637 PMID: 10587496

61. Tsukatani T, Miwa T, Furukawa M, Costanzo RM. Detection thresholds for phenyl ethyl alcohol using serial dilutions in different solvents. Chem Senses. 2003; 28: 25–32. https://doi.org/10.1093/chemse/28.1.25 PMID: 12502521

62. Laska M. Olfactory Discrimination Learning in an Outbred and an Inbred Strain of Mice. Chem Senses. 2015; 40: 489–496. https://doi.org/10.1093/chemse/bjv032 PMID: 26123553

63. Slotnick B, Schellinck H. Methods in olfactory research with rodents. Frontiers and methods in chemosenses. Boca Raton, FL: CRC; pp. 21–61.

64. Laska M. Olfactory Discrimination Ability of Human Subjects for Ten Pairs of Enantiomers. Chem Senses. 1999; 24: 161–170. https://doi.org/10.1093/chemse/24.2.161 PMID: 10321817

65. Feng G, Zhou W. Nostril-specific and structure-based olfactory learning of chiral discrimination in human adults. eLife. 2019; 8: e41296. https://doi.org/10.7554/eLife.41296 PMID: 30652684

66. Sato T, Kobayakawa R, Kobayakawa K, Emura M, Itohara S, Kizumi M, et al. Supersensitive detection and discrimination of enantiomers by dorsal olfactory receptors: evidence for hierarchical odour coding. Sci Rep. 2015; 5: 14073. https://doi.org/10.1038/srep14073 PMID: 26361056

67. Laska M, Shepherd GM. Olfactory discrimination ability of CD-1 mice for a large array of enantiomers. Neuroscience. 2007; 144: 295–301. https://doi.org/10.1016/j.neuroscience.2006.08.063 PMID: 17045753

68. Fleischmann A, Shykind BM, Sosulski DL, Franks KM, Glinka ME, Mei DF, et al. Mice with a "monoclonal nose": perturbations in an olfactory map impair odor discrimination. Neuron. 2008; 60: 1068–1081. https://doi.org/10.1016/j.neuron.2008.10.046 PMID: 19109912

69. Cometto-MuñIz J. Trigeminal and Olfactory Chemosensory Impact of Selected Terpenes. Pharmacol Biochem Behav. 1998; 60: 765–770. https://doi.org/10.1016/s0091-3057(98)00054-9 PMID: 9678663

70. Clarin T, Sandhu S, Apfelbach R. Odor detection and odor discrimination in subadult and adult rats for two enantiomeric odorants supported by c-fos data. Behav Brain Res. 2010; 206: 229–235. https://doi.org/10.1016/j.bbr.2009.09.022 PMID: 19766144

71. Cometto-Muñiz JE, Cain WS. Thresholds for odor and nasal pungency. Physiol Behav. 1990; 48: 719–725. https://doi.org/10.1016/0031-9384(90)90217-r PMID: 2082372

72. Dunkel A, Steinhaus M, Kotthoff M, Nowak B, Krautwurst D, Schieberle P, et al. Nature's Chemical Signatures in Human Olfaction: A Foodborne Perspective for Future Biotechnology. Angew Chem Int Ed. 2014; 53: 7124–7143. https://doi.org/10.1002/anie.201309508 PMID: 24939725

73. Shepherd GM. The Human Sense of Smell: Are We Better Than We Think? PLoS Biol. 2004; 2: e146. https://doi.org/10.1371/journal.pbio.0020146 PMID: 15138509

74. McGann JP. Poor human olfaction is a 19th-century myth. Science. 2017;356. https://doi.org/10.1126/science.aam7263 PMID: 28495701

75. Sarrafchi A, Odhammer AME, Hernandez Salazar LT, Laska M. Olfactory sensitivity for six predator odorants in CD-1 mice, human subjects, and spider monkeys. PloS One. 2013; 8: e80621. https://doi.org/10.1371/journal.pone.0080621 PMID: 24278296

76. Laska M. Human and Animal Olfactory Capabilities Compared. In: Buettner A, editor. Springer Handbook of Odor. Cham: Springer International Publishing; 2017. pp. 81–82. https://doi.org/10.1007/978-3-319-26932-0_32

77. Wackermannová M, Pinc L, Jebavý L. Olfactory sensitivity in mammalian species. Physiol Res. 2016; 65: 369–390. https://doi.org/10.33549/physiolres.932955 PMID: 27070753

78. Magan N, Evans P. Volatiles as an indicator of fungal activity and differentiation between species, and the potential use of electronic nose technology for early detection of grain spoilage. J Stored Prod Res. 2000; 36: 319–340. https://doi.org/10.1016/s0022-474x(99)00057-0 PMID: 10880811

79. Pellizzon MA, Ricci MR. Choice of Laboratory Rodent Diet May Confound Data Interpretation and Reproducibility. Curr Dev Nutr. 2020; 4: nzaa031. https://doi.org/10.1093/cdn/nzaa031 PMID: 32258990

80. Dewan A, Pacifico R, Zhan R, Rinberg D, Bozza T. Non-redundant coding of aversive odours in the main olfactory pathway. Nature. 2013; 497: 486–489. https://doi.org/10.1038/nature12114 PMID: 23624375

81. Ferrero DM, Lemon JK, Fluegge D, Pashkovski SL, Korzan WJ, Datta SR, et al. Detection and avoidance of a carnivore odor by prey. Proc Natl Acad Sci U S A. 2011; 108: 11235–11240. https://doi.org/10.1073/pnas.1103317108 PMID: 21690383

82. Silvestre AJD, Gandini A. Terpenes: Major Sources, Properties and Applications. Monomers, Polymers and Composites from Renewable Resources. Elsevier; 2008. pp. 17–38. https://doi.org/10.1016/B978-0-08-045316-3.00002–8

83. Frost CJ, Appel HM, Carlson JE, De Moraes CM, Mescher MC, Schultz JC. Within-plant signalling via volatiles overcomes vascular constraints on systemic signalling and primes responses against herbivores. Ecol Lett. 2007; 10: 490–498. https://doi.org/10.1111/j.1461-0248.2007.01043.x PMID: 17498148

84. Badan D. Diet of the house mouse (Mus musculus L.) in two pine and a kauri forest. N Z J Ecol. 1986; 9: 137–141.
